# The spread of agriculture in Iberia through Approximate Bayesian Computation and Neolithic projectile tools

**DOI:** 10.1371/journal.pone.0261813

**Published:** 2021-12-28

**Authors:** Alfredo Cortell-Nicolau, Oreto García-Puchol, María Barrera-Cruz, Daniel García-Rivero

**Affiliations:** 1 Departament de Prehistòria, Arqueologia i Història Antiga, Facultat de Geografia i Història, Universitat de València, València, Spain; 2 Department of Archaeology, McDonald Institute for Archaeological Research, Faculty of Human, Social, and Political Science, University of Cambridge, Cambridge, United Kingdom; 3 Department of Prehistory and Archaeology, Faculty of Geography and History, University of Seville, Seville, Spain; University at Buffalo - The State University of New York, UNITED STATES

## Abstract

In the present article we use geometric microliths (a specific type of arrowhead) and Approximate Bayesian Computation (ABC) in order to evaluate possible origin points and expansion routes for the Neolithic in the Iberian Peninsula. In order to do so, we divide the Iberian Peninsula in four areas (Ebro river, Catalan shores, Xúquer river and Guadalquivir river) and we sample the geometric microliths existing in the sites with the oldest radiocarbon dates for each zone. On this data, we perform a partial Mantel test with three matrices: geographic distance matrix, cultural distance matrix and chronological distance matrix. After this is done, we simulate a series of partial Mantel tests where we alter the chronological matrix by using an expansion model with randomised origin points, and using the distribution of the observed partial Mantel test’s results as a summary statistic within an Approximate Bayesian Computation-Sequential Monte-Carlo (ABC-SMC) algorithm framework. Our results point clearly to a Neolithic expansion route following the Northern Mediterranean, whilst the Southern Mediterranean route could also find support and should be further discussed. The most probable origin points focus on the Xúquer river area.

## Introduction

The Neolithic arrival at the Iberian Peninsula has been explained through a mixed model triggered by the demic expansion across the Mediterranean. At this point, the seminal works of Ammerman and Cavalli-Sforza [1, 2] constitute the basis of the current proposals considering the continental scale. New arguments have also been provided by ancient DNA [3, 4] confirming the main demic character of the Neolithic expansion linked with the demographic success as a result of the agricultural way of life [5–7]. Since then, some authors have highlighted the important role played by the coastal dispersal and the mobility of the first farmers [8–11], this assumption offering a good fit —empirical and modelled— according to current C14 dates [12]. In a general overview, and referring to the coastal Mediterranean expansion, the northern path is considered the main route taking into account several mechanisms that can involve “leapfrog” movements [10], although the southern route around the northern African coast has also been discussed [10, 13, 14]. In this sense, cultural evolutionary trajectories offer some valuable hints for contributing to understand different archaeological patterns and processes. This kind of approach reveals interesting results in order to investigate cultural transmission processes in the context of population dispersal [15]. Focusing on the Western Mediterranean, recent studies addressing decoration techniques in the Neolithic pottery record have been used to investigate patterns and mechanisms of the agricultural spread [16, 17]. The promising results achieved so far reinforce the scattered and discontinuous character of the Neolithic expansion, while also providing evidence of some kind of branching phenomena [18]. In this paper, and through a computational analysis of stylistic diversity, we use for the first time geometric microliths to explore cultural variability at the times of the pioneer arrival of the Neolithic at the Mediterranean coast of Iberia around the middle of the VIII millennium cal BP. The final goal is to understand, given the similarity patterns involved, the most likely origin points and routes of arrival for the first farmers in the Iberian Peninsula. The rationale is to compare chronological possibilities for the Neolithic spread, given the geographic distance and cultural similarity of the different assemblages. Thus, in this context, we aim to investigate how the observed patterns in cultural distances could reveal differences regarding the chronological distribution of the first Neolithic groups in the Iberian Peninsula. Particularly, we will characterize the dispersal character and trajectories considering three possible routes: northern Mediterranean, inner Pyrenees and southern (northern Africa) routes. To do this we have designed our research so that it: 1) compiles a geometric tools dataset from the first Neolithic dated settlements in a wide region from the northeast to the Guadalquivir valley, 2) performs statistical analyses based on cultural distance matrices and Bayesian computational simulation and 3) explores the results evaluating cultural diversity from spatial parameters and mechanisms of population dispersal including the northern and southern pathways.

## An appraisal of the Neolithic spread through the mediterranean Iberia

The Neolithic package, including domestic plants, animals and cultural items as impressed pottery ware, polished stone and sickles among others was introduced in the Iberian Peninsula circa 7600 cal BP. Its fast dispersal around the Mediterranean corridor reflects the punctuated appearance of some pioneering areas from the northeast to the southwest. Current radiocarbon records confirm the closeness between the oldest dates at the coastal territory and some sites in the Ebro valley considering dates from domestic samples [5]. Furthermore, ancient DNA analysis has highlighted the demic signal of the expansion in the northeast (Cova Bonica) and the eastern region (Cova de la Sarsa) [3, 4, 19] and also reveals some kind of admixture at larger territorial scales [4]. In this scenario the Iberian Peninsula offers a range of situations characterised by a low and irregular presence of the late hunter-gatherer population, which concentrates in the coastal territories excepting Catalonia and Andalusia, and shows a general emptiness for the Meseta region.

In a brief appraisal, from the Ebro river to roughly the Segura river there is evidence of a Mesolithic record belonging to the blades and trapezes technocomplex, also extended along the Ebro river and the Atlantic coast of Portugal [5, 20–24], which appears in the Mediterranean Iberia around 8600 cal BP without previous cultural tradition. Here, the occurrence of the latest Mesolithic inhabitants has been described especially in the Low Ebro valley and the eastern shores including the provinces of Castelló, València and Alacant. Interestingly, at the beginning of the VIII millennium cal BP, the Mesolithic settlements seem to relocate, at least in some coastal areas, and perhaps triggered by the start of the Neolithic advance in the central Mediterranean. This reorganisation will define the arrival landscape of the first Neolithic settlers in the area.

**Fig 1:**
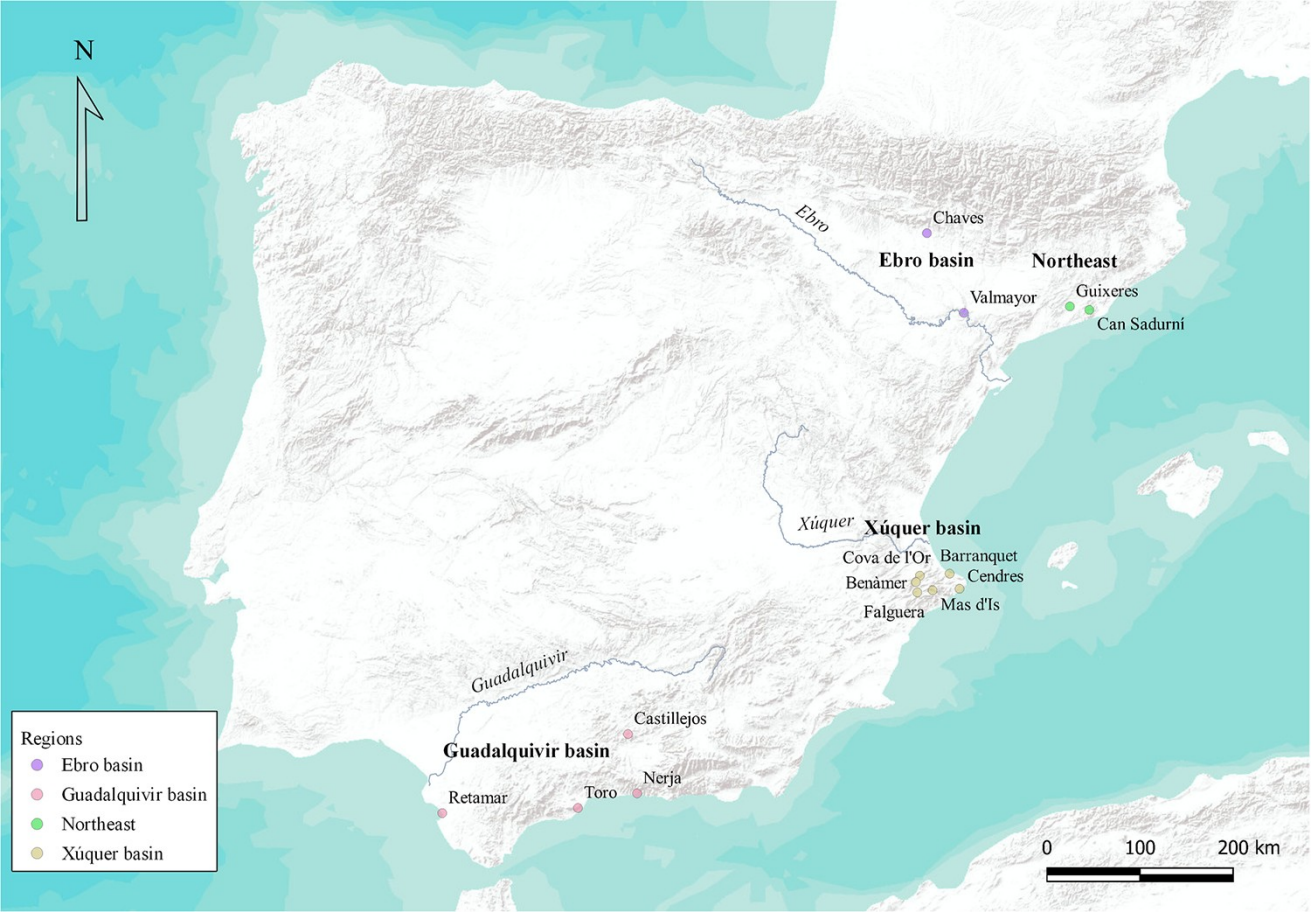
Sites and areas considered for this study. Only the sites containing geometric microliths for the time-span under study are shown. Maps modified from ESRI World Terrain Base Map.

From the North to the South, it is possible to recognise some Neolithic pioneering areas in Catalonia, the middle Ebro river, the Serpis valley/Cap de la Nao region, and the coast of Málaga and Cádiz, coincident with areas with a scarce or non-existent Mesolithic population (Fig 1). Impressed pottery constitutes the characteristic record of the first Neolithic sites including *cardial* impressions among other decorations. Relating to geometric projectiles we can observe some degree of diversity which will be analysed later.

Regarding the phenomenon of the first *impressa* pottery decorated with “sillon d’impressions”, it has been recently individuated at the site of El Barranquet (Oliva, València) [25, 26], and is also present at sites like Mas d’Is (Penàguila, Alacant) [27, 28]. However, and unlike the situation described for Southern France [29], the current radiocarbon dataset does not allow us to distinguish a specific phase. From this point, the archaeological record reflects different situations. In the northeast we can observe a fast spread of the Neolithic way of life mainly in the territory located between the Llobregat near to Barcelona and the Daurada coast in Tarragona [30]. Several open-air sites and cave occupations have been identified conforming the start of the Neolithic way of life in an area without clear signals of Mesolithic inhabitants.

The Cueva de Chaves (Bastarás, Huesca) constitutes a pioneer site that opens the question of the advance to the inner Iberia through the Pre-Pyrenees mountains from the Mediterranean coast [31]. The Neolithic package here includes domestic plants and animals and also a characteristic Neolithic record showing cardial decorated pottery and other typical Early Neolithic tools made from bones and knapped cherts. Other sites, such as Valmayor, located in the Low Aragon area, have been pointed out as the result of an acculturation process from the Mesolithic on the basis of the absence of domestics and the concurrency of late Mesolithic groups in its proximity [32]. Nevertheless, some authors do not discard their Neolithic attribution considering their cultural assemblage and chronological framework (from level II) [23]. As we can see, a more isolated character could be attributed to these first Neolithic pioneer sites considering current data in the Ebro valley until the last centuries of the VIII millennium cal BP [33].

Moving to the eastern shore of the Iberian Peninsula, the area between the mouth of the Serpis river and the Cap de la Nau, including the Serpis valley from its headwater, constitutes a genuine core area for the Neolithic dispersal. The sites of Cova de les Cendres, Barranquet, Cova d’En Pardo, Cova de l’Or, Benàmer, Mas d’Is, Falguera and Cova de la Sarsa report radiocarbon dates centred around 7500 cal BP [34–36]. The recognition of open-air sites and the use of caves and small rock-shelters present a structured spatial pattern devoted to the new agricultural way of life, also reflected in different symbolic markers across the territory, interpreted as some kind of sanctuaries (Petracos and La Sarga). The fast expansion around the Serpis valley would point to an earlier time of the arrival, although this aspect is not currently confirmed.

From the south, a more punctuated dispersal record is registered in a long territory from the Segura river to the Cádiz coast in Andalusia [37–40]. Although a significant number of early Neolithic sites is known, the oldest dates concentrate in a small number of them, located in the coast of Málaga (Cueva de Nerja), the inner of Málaga (Cueva del Toro), Granada (Castillejos and Carigüela) and Cádiz (Cueva de Dehesilla), where pottery with *impressa* decoration may have also been found [41]. The bulk of information corresponds with the occupation of caves even though open-air sites have been also identified [42]. The site of Retamar, attributed of the Early Neolithic [43] seems to present a dichotomous nature as has been discussed by Zilhão [8]. The current radiocarbon dataset confirms the rapidity of the arrival of the Neolithic at the western Andalusian coast.

In a general overview, the model postulated by Zilhão [8] relating with the “Maritime pioneer colonization” is the most fit according to the current chronological framework. The modelling approach proposed by Isern et al. [12] considers a leapfrog movement with a minimum jump of 300 km per generation that reveals a front of advance of 5 km per year from the Mediterranean coast to the Atlantic shores of Portugal, while also pointing that cross-mating with previous Mesolithic inhabitants, acculturation and mutual interaction could increase the rate of spread. Focusing on Mediterranean Iberia, this kind of situation has been described in the Valencian region and the Ebro valley by the so-called “Dual model” [44–47], expanded from its original application in the pioneer Neolithic of the Cap de la Nao/Serpis valley and the surrounding Mesolithic areas [23]. According to this model, farming arrives to the Iberian Peninsula along with the Neolithic people expanding throughout the Mediterranean. Hence, the duality of the model relies on the fact that, from this initial demic contribution, the agricultural practices can be brought either (1) by the newcomers without contact with the previous Mesolithic population or (2) they can be fruit of the interaction between those Neolithic newcomers and the previous hunter-gatherer inhabitants. Despite the difficulties for disentangling acculturation processes in the archaeological record, this proposal remains the best scenario according with the current data.

Consequently, to look over evolutionary processes behind cultural variability at the times of the Neolithic expansion becomes a challenge for exploring patterns and processes in cultural change. In this paper we turn to geometric tools considering the potential exhibited by stylistic traits in order to account for cultural change [15, 48].

## Methodology

### Data

The potential of stylistic traits for similarity and culture transmission analysis has been noted by several authors [49–51]. Although from the beginning of the Neolithic, some of the most significant studies focus on the variability of pottery (decoration patterns and/or shapes) and ornaments [17, 52–54], the literature concerning stylistic lithic variation approaches, from an evolutionary perspective, also displays some promising works [15, 48, 55–57]. In our case we put the focus on geometric projectiles belonging to the first Neolithic, also able to reflect cultural variability.

#### Sites

The first step has been to sample the most adequate sites in order to capture the early moments of the Neolithization process in the Iberian Peninsula. With this scope, we have divided the zone under study in four different areas from which we have selected the oldest radiocarbon date from short life single samples provided by domestic plants or animals around the middle of the VIII millennium cal BP. The zones selected loosely correspond with current Aragon, Calatonia, Valencia and Andalusian regions, while, speaking broadly, they also have their correspondence with the Ebro, northeast shores, Xúquer and Guadalquivir fluvial basins respectively. The oldest dates for each zone belong to the sites of Chaves [58], Guixeres [30], Mas d’Is [27] and Cueva de Dehesilla [40], respectively. Once the first date has been selected, other sites belonging to the same area have also been sampled according to the following criteria: (1) they must testify the presence of Early Neolithic assemblages and (2) their oldest calibrated radiocarbon date must overlap to any extent with the oldest calibrated radiocarbon date proposed for the area. After applying this filter, we have remained with a total of 14 sites, which satisfy the general premises. However, some of the sites deserve special comments. In this sense we have included the site of Valmayor [32] for the Ebro Valley, considering the possibility of its Neolithic attribution [36]. On the opposite, we also introduce the site of Retamar [59], despite the discussion regarding its interpretation and the chance that it may actually be representing an admixture of Mesolithic and Neolithic occupations [60].

#### Geometric projectiles

**Fig 2:**
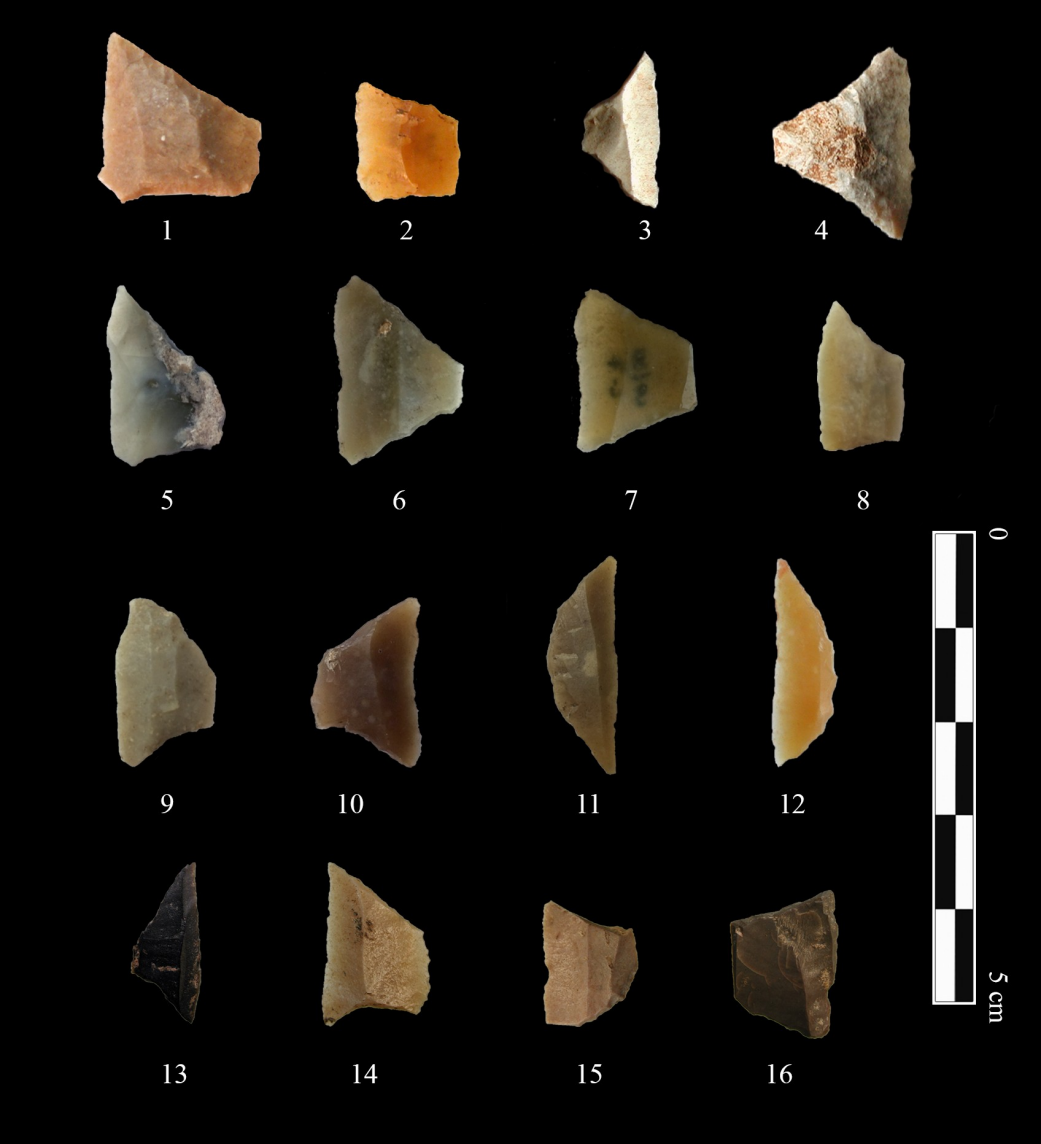
Some of the geometric microliths present in the sites under study. The geometrics belong to the sites of Cova de les Cendres (1,2), Barranquet (3,4), Cova de l’Or (5–11), Mas d’Is (12) and Cueva de Nerja (13–16). Full information of the geometric microliths used (including their provenance) can be found in S2 File in Geos.csv and Specimen_information.csv. In this figure, geometrics 1 and 2 are republished from [72] under CC BY license, with permission from MARQ–Museu Arqueològic d’Alacant, original copyright 2009. Likewise, geometrics 3, 4 and 12 are republished from [28] under CC BY license, with permission from A. Cortell-Nicolau and O. García-Puchol, original copyright 2020.

The geometric microliths are a specific type of arrowhead used both by the communities of the last hunter-gatherers and the first farmers of the Iberian Peninsula (Fig 2). Typological classifications have been traditionally used from very different approaches in order to systematise and understand the meaning of cultural evolution and grouping in lithic industry, also applied to this specific type of arrowhead ([61–63] to name some examples). In the Iberian Peninsula, one of the most widely used classifications for the Holocene industries has been the one of Fortea [64] which, broadly speaking, classifies the geometric microliths in three groups; trapezes, triangles and circle segments, including different subgroups adding up to 18 different types (see also [22] for a complete review of the geometrization of the Iberian Peninsula during the Late Mesolithic). Conversely, from the last quarter of the 20th century some authors started noting the possible problems attributed to a somehow arbitrary classification of archaeological types [65]. In this sense, an alternative approach, based on the construction of paradigmatic classes was proposed [65]. This approach has been further enlarged and refined with the contribution of other authors, and it consists mainly in the development of a hyper-space defined by single-trait selection, where the presence of a character state is exclusive of the presence of another. This creates a series of taxa, which has been widely used in different types of studies, from cultural phylogenies to cultural transmission models [66–71].

In this work, under the assumption that stylistic traits can account for cultural variance (see [51] for an extensive discussion), we have decided to focus on the single trait rather than grouping by different types and/or taxa. The rationale is that, regardless of how the grouping is produced, as soon as an archaeological item is categorised by assigning it to one archaeological type (or taxon) an artificial segregation, whose significance is unknown to current archaeology, is already being established. In this sense, we have decided to work directly at single-trait level, trying to let the data *speak for itself*. In this matter, geometric microliths are valid proxies to test for evolutionary stylistic transmission, as their use as arrowheads has been widely stated by archaeological literature [73–80], despite possible complementary uses [75, 81]. Furthermore, and in order to ensure the stylistic character of the procedure, we have removed every geometric whose main use may be related to agriculture such as clear sickles (either because of their polish or their shape). In this way, we can consider the same use for the tools under study, thus releasing them from possible biases due to evolutionary drifts responding to functional pressure.

The collection of data traits has been performed automatically (while every geometric microlith has been personally supervised to ensure the consistency of the method). In order to do so, we have used the R package GeomeasuRe [82, 83]. This package is able to capture different kinds of morphometric measures from vectorised images. These include the most widely used measures, such as angles, side length, symmetry or ratios while also including different types of reliability measures and the so-called L-measures. Thus, the output is a series of lines of different lengths able to capture the morphometry of the geometric. All in all, we have captured a total of 233 L-lines complemented with different measurements, such as total length, total width, area of the geometric, angle and the factor variables retouch direction (for distal and for proximal side) and retouch mode (for distal and proximal side). To all this we must add different kinds of reliability measures, accounting for the completeness of the geometric.

In order to avoid size problems derived from different raw material sources, the geometrics have been scaled, after which we have filtered the data following three main steps: (1) removing the geometric microliths which did not meet the reliability criteria, (2) removing the L-lines without presence of the geometric and (3) performing a PCA in order to reduce the dimensionality of our dataset. After obtaining the values in the filtered data of the PCA, we have categorised our numeric data, and added categoric traits relating with retouch mode and direction, in order to obtain values of representation of the trait for each site. Since the site of Can Sadurní had only one geometric, and it did not meet the reliability criterion, this site has been removed from our study. All in all, we have remained with a total of 13 sites and 146 geometric microliths, accounting for 19 variables (data in S2 File in Variables_after_PCA.CSV). These operations are explained at length in S1 File.

### Statistical analysis

As explained above, our main goal is to understand the cultural similarities of the geometric microliths from the sites under study and, based on those similarities, evaluate different possible origin points and routes of expansion for the Neolithic in the Iberian Peninsula. In order to do so, we have relied on Mantel Tests and Approximate Bayesian Computation, more specifically Sequential Monte-Carlo algorithms (SMC-ABC) [84, 85]. Mantel Tests have been commonly used in archaeology in order to detect cultural similarities [15, 86, 87]. This type of test measures the relationship between two distance matrices, defined as A and B for the example, being the null hypothesis that distances of matrix A are independent of distances of matrix B. To do this, a randomization test assesses the significance of the association between the two matrices (see [88] for a more extended discussion). In order to avoid possible type I errors due to spatial auto-correlation [89], we have performed Moran’s I tests on each variable, confirming the inexistence of spatial auto-correlation (see S1 File for a more detailed discussion on the use and appropriateness of Mantel tests, both general and for this specific work).

The rationale behind partial Mantel tests is similar to the one above, but where a third matrix is used in order to control for the other two. In this sense, this third matrix is held constant while the relationship between the other two is determined [90]. Thus, we have three different matrices: (1) Matrix A accounts for cultural distances; (2) Matrix B measures the Euclidean distance between each site, and (3) Matrix C contains the oldest short-live Neolithic radiocarbon date for each site. The key to this work is that, while holding matrices A and B constant, we perform a series of simulations on possible expansion routes, considering randomly different origin points. Each of these simulations will produce a new simulated chronological matrix (simulated Matrix C), which will give a different result in the partial Mantel test. Then, the similarity results yielded from these combined matrices are finally compared to the similarity results observed in the current archaeological record, in order to obtain a controlled environment when we can actually track possible origin points and routes. Focusing on the chronological matrix, because we are working on calibrated dates, choosing one single date to represent a site can be tricky. One option is to choose an average of the calibrated date [53], while for the construction of diachronic models, more sophisticated options, such as the use of Bayesian hierarchic models have been proposed [91]. However, and accounting for the fact that in this case we are focusing on a single calibrated date per site, we have decided to sample the calibrated date from the probability distribution of the calibration of the BP date. We are aware that this procedure might introduce randomness to the process, but (1) with enough number of simulations, the dates should converge to their most probable value and (2) when and if the dates selected are too far away from their most probable values the test will return non-significant or extreme results, which will later be either discarded in the first case, or filtered in the second, as extreme points in the probability distribution will not be used in the ABC process. The data has been calibrated using the R package rcarbon [92].

Finally, and because Mantel tests focus on population structure, it has also been noted whether they can actually account for cultural diversity and affiliation, proposing some possible corrections [53]. Although this is something worth keeping in mind, we have decided to assume them as a valid proxy, as is common in cultural diversity studies [88, 93, 94], leaving further refinement open for discussion. In any case, and having this into account, previous simulations have been performed in order to test the validity of the method, as stated in S1 File.

#### The model

As briefly mentioned above, we have used the third matrix (the chronological Matrix C) added to partial Mantel tests to control for possible expansion routes. Reminding that, in our case, this third matrix contains the oldest short-live radiocarbon date for each site, and because due to the permutation process the values of the Mantel-t statistic are not always the same, we have created a distribution of n = 1000 significant Mantel t values of our data. This distribution will be used as the observed summary statistic in the SMC-ABC process (see next paragraph). After obtaining the summary statistic, we modify the 14C matrix in order to propose different points and modes of expansion. In order to do so, we randomly select one possible starting point from the 13 sites under study and assign its actual 14C dating (calibrated in the way explained above). Because we are not considering a very large geographic area, we have decided to use the actual sites rather than possible simulated origin points, as it has been proposed for larger areas [95]. After that, we have considered a simple expansion model, defined as:

$$y_{i}=t-\frac{o_{i}}{r}$$

Being *y* the vector with the modelled dates for each site, *t* the calibrated date of the site randomly chosen as starting point, *o* the vector of distances per site and *r* the expansion rate. The rationale is that, because the other two matrices (geographic distance and cultural similarities) are held equal for each iteration, the different chronological proposals, should be able to produce, as a result, a series of Mantel-t values, whose distribution should approach the values of the real data, and where expansion patterns can be inferred.

The model has been applied to one or two possible origins. For the case of the two origins, the model remains the same for each origin (also selecting the actual 14C dating to each of the two sites). In this case, the two origins would produce a possible *arrival date* for each site. Thus, for each site not stated as the possible origin, we keep the oldest date attributed from each starting point.

#### SMC-ABC approach

The Approximate Bayesian Computation approach [96] has become popular in the last years to deal with Bayesian inference where the likelihood function is impossible or expensive to achieve (see [71, 87, 97–103] for applications of the method in archaeology and culture evolution). It relies on simulation in order to create posterior distributions from the data. The basic rationale consists of the creation of a summary statistic, here defined as *ϵ*, which will be the basis on which simulations will be accepted or rejected. This statistic *ϵ* needs to be a value which can be derived both from the observed and the simulated data. Nevertheless, *ϵ* is not a general predefined value and must be constructed *ad hoc* by the researcher depending on the data (see [104, 105] for detailed discussions on summary statistics). Once *ϵ* has been established, an acceptance threshold is defined and only the simulations meeting the criteria of the threshold will be accepted. There are several ways to do this (see [98]). The most basic method relies on the so-called rejection algorithm, by which a certain number of simulations below a pre-defined threshold are accepted. This algorithm, however, is usually expensive, as it may require a large number of simulations. Thus, in order to speed up the process, some alternative ways have been proposed. One of them includes the use of Markov-Chain Monte Carlo [106]; and the one used here is based on the so-called Sequential Monte-Carlo methods. This algorithm relies on the construction of particles *θ*_*i*_ [84]. First a simple rejection algorithm is developed, where a number of simulations is accepted in order to form the first posterior distribution. This becomes the first particle *θ*_1_. Then, this posterior distribution *θ*_1_ becomes the prior distribution for the next particle *θ*_2_, and this operation is repeated *n* times for a good convergence of the posterior. As a difference to the other methods, when each particle *θ*_*i*_ is accepted and becomes the prior distribution for the next iteration, the threshold proposed for *ϵ* is reduced, which brings a better convergence for the posterior. More specifically, in order to create the current particle *θ*_*i*_, the algorithm samples one observation from the previous particle *θ*_*i*−1_, which becomes the candidate for acceptance under this simulation. The next step is to modify the parameters of the candidate observation by a distribution kernel, frequently ~*U*(−1,1) [85]. After the simulation is performed, the threshold between *s*_*o*_ (the observed summary statistic) and *S*_*s*_ (the simulated summary statistic) is evaluated again, and accepted only if it improves the results of the previous particle. Because the threshold value is smaller for each *θ*_*i*_, each particle provides a better convergence regarding the prior than the previous one.

We have used this method also due to the randomness introduced when selecting each calibrated radiocarbon date, taking into account the increase in computational efficiency. We have used one first rejection algorithm, and three more particles, where *θ*_*i*_ = 1000 observations. Because we deal with the distributions of the Mantel-t, we do not have a single *S*_*o*_ value to compare with. Thus, for the initial rejection algorithm, we have accepted only significant values falling within the (*Q*_1_, *Q*_3_) from *S*_*o*_. For the next three particles, the simulations accepted range between (*P*_30_, *P*_70_), (*P*_35_, *P*_65_) and (*P*_40_, *P*_60_). In order to compare different possibilities, we have decided to use two transition kernels, a strict transition kernel $∼U(\frac{r}{100}\times 90,\frac{r}{100}\times 110)$ where the pre-selected *r* clearly conditions the *r* parameter for the simulation, and ~*U*(*r*−1, *r*+1) where there is more freedom for the selection of *r* in the new simulation. As for the priors, for each starting date, we have used the probability of the calibrated date, as explained above, whereas the parameter *r* follows in any case a ~*U*(1,5) distribution, in agreement with current archaeological literature [12].

The nuances of these methods are explained in more detail at S1 File.

## Results

We have constructed *S*_*o*_ from the results of the permutations on the chronological matrix of the Mantel test. In this sense, we have obtained a distribution from the significant values, accounting for a dissimilarity measure with a mean of 0.37 and the thresholds 0.23–0.51 for a 95% confidence interval under a normal distribution, thus being able to distinguish significant differences among the sites under study. These values have been the key which the posterior analyses have relied on, as they are used as the ‘target’ similarity measure for the simulation process. Two possible hypotheses have been considered, one where the Neolithic would have a single origin area in the Iberian Peninsula, and another one where there would be two possible synchronic–*sensu lato–*origin areas.

### Single-origin

**Fig 3:**
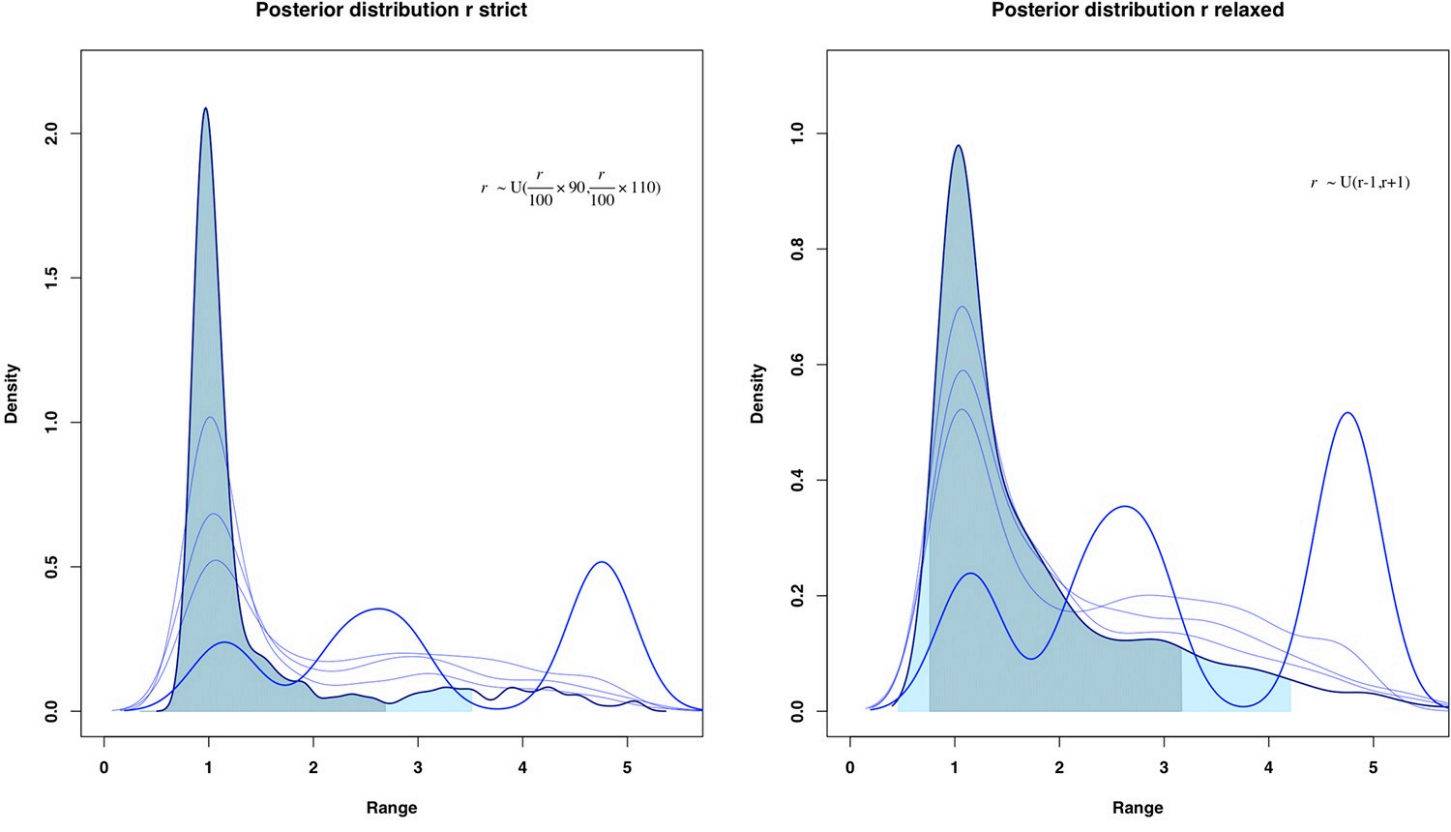
Convergence of the posterior distribution of the parameter *r*. Confidence intervals offered at 80% and 95%. For both kernels, from the initial uniform distribution, the parameter value offers better convergence for each *θ*_*i*_.

After the development of the process, one of the first outputs calling our attention is the fact that the distribution of the parameter *r* skews clearly towards 1, with HDP 95% of 0.45–3.51 for the restricted kernel and 0.46–4.21 for the relaxed kernel, considering in both cases a Gamma distribution (Fig 3). On one hand, this is indicative of a good convergence of the posterior. On the other hand, and although this is not the central objective of this work, we could note very shortly that this agrees with the general expansion values originally proposed by Ammermann and Cavalli-Sforza [2]. Of course, there would probably be regional differences and trends [12], which could also be responsible for the wide confidence interval, but which will not be studied here. Taking the Iberian Peninsula as a large space, we could resume that *r* values approach to a credible interval.

**Fig 4:**
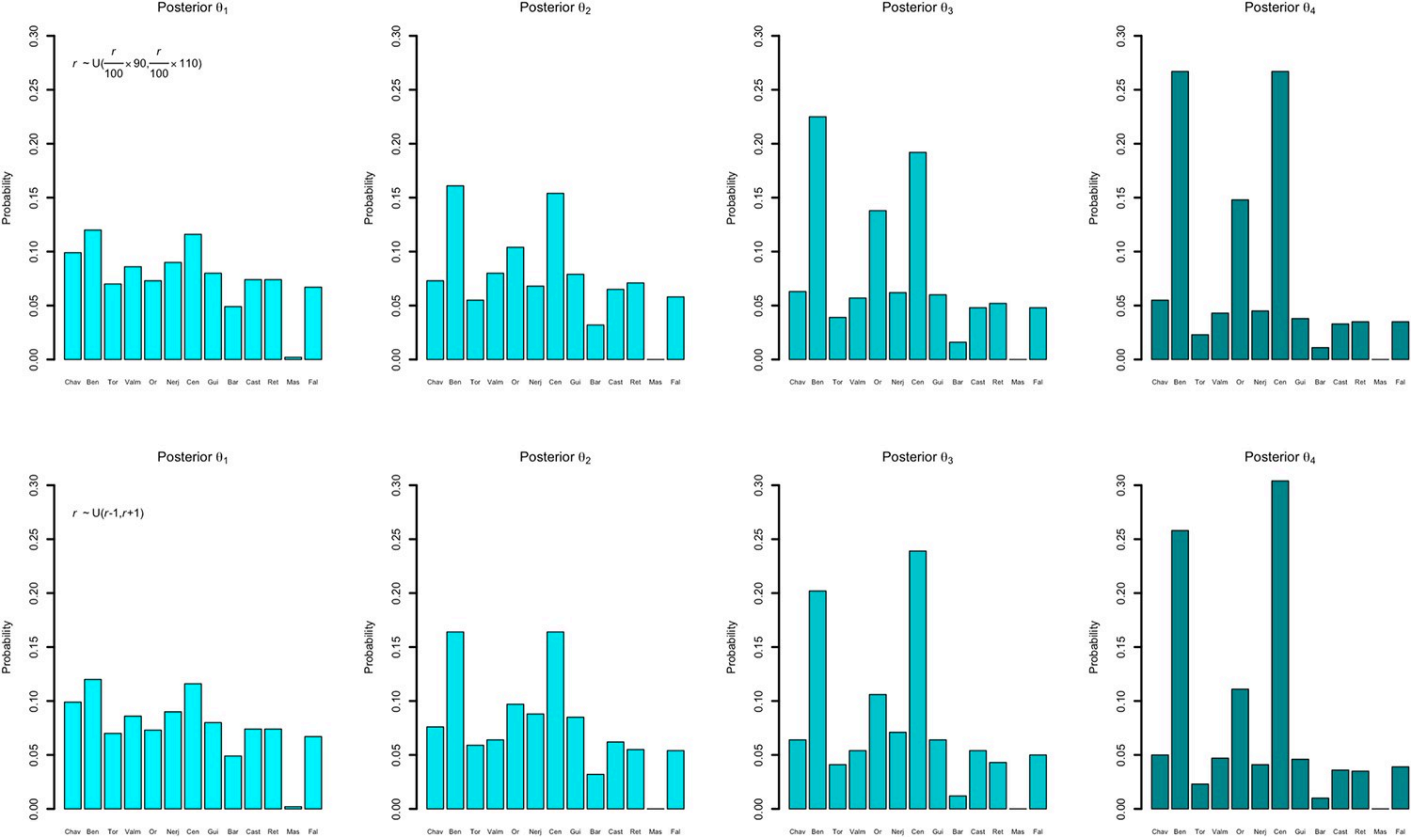
Posterior distribution of the possible origins for each particle. Site abbreviations from left to right: Chav = Chaves, Ben = Benàmer, Tor = Cueva del Toro, Valm = Valmayor XI, Or = Cova de l’Or, Nerj = Cueva de Nerja, Cen = Cova de les Cendres, Gui = Guixeres, Bar = Barranquet, Cast = Los Castillejos, Ret = El Retamar, Mas = Mas d’Is, Fal = Abric de la Falguera.

Our variable of interest is the one containing the origin points for each simulation. In this sense, if we look at Fig 4, we can see that there is almost no difference between choosing a more restrictive or a more relaxed kernel. In both cases, the most probable sites remain Benàmer and Cova de les Cendres, with a very small difference between them (these two sites are, in any case, very close in space and time), and a probability of 26.7% each in the case of the restrictive kernel and 25.8% for Benàmer and 30.4% for Cendres in the case of the relaxed kernel. Also, and although this has already been noted for Mantel tests [88] sample size at site-level has not played any role, as the sites with the highest presence of geometric microliths (Retamar and Cova de l’Or) show probabilities of 3.5% for both kernels in Retamar and 14.8% for the restricted kernel and 11.1% for the relaxed kernel in Or, where 2×P(*x*_*ret*_)<P(*Mo*) in the first case and clearly P(*x*_*or*_)<P(*Mo*) in the latter (also 2×P(*x*_*or*_)<P(*Mo*) for the case of the relaxed kernel).

**Fig 5:**
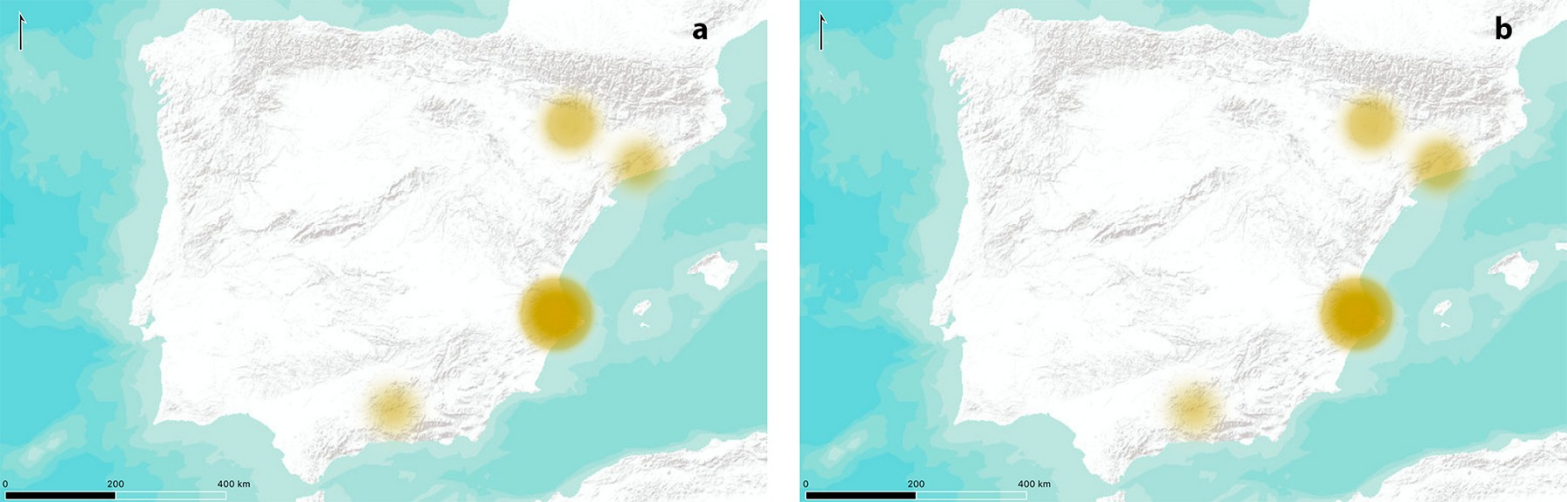
Probability for the proposed origin areas considering a single origin. (A) relaxed kernel. (B) strict kernel. Higher intensity and size of circles indicates higher probability. Maps modified from ESRI World Terrain Base Map.

These specific results cannot be interpreted in a strict sense, but rather they would account for spread signals regarding each area. Thus, we have considered these as possible origins and, mainly, as aggregated values. We have done this in two ways; first, we have considered regional divisions attending to fluvial basins, which broadly coincide with current regional administrations, as stated in the methods section, and second, we have considered three possible expansion routes. In the first case, we consider four possible nuclei; the Aragon nuclei (Ebro basin), the Catalan nuclei (Northeast basin), the Valencian nuclei (Xúquer basin) and the Andalusian nuclei (Guadalquivir basin). In order to avoid problems due to sample size (as some nuclei have more sites than others), we have applied a correction where the total aggregated probability is divided by the number of sites producing that probability. After performing these operations, we can observe how the nuclei of Xúquer basin offers the highest probability (50%), followed by Ebro basin, Northeast and Guadalquivir basin, all of them offering similar probabilities (20.25%, 15.7% and 14.05% respectively) when we consider the restrictive kernel, whereas the results do not vary substantially for the case of the relaxed kernel (48.39%; 19.35%; 18.55%; 13.71%) (Fig 5).

**Fig 6:**
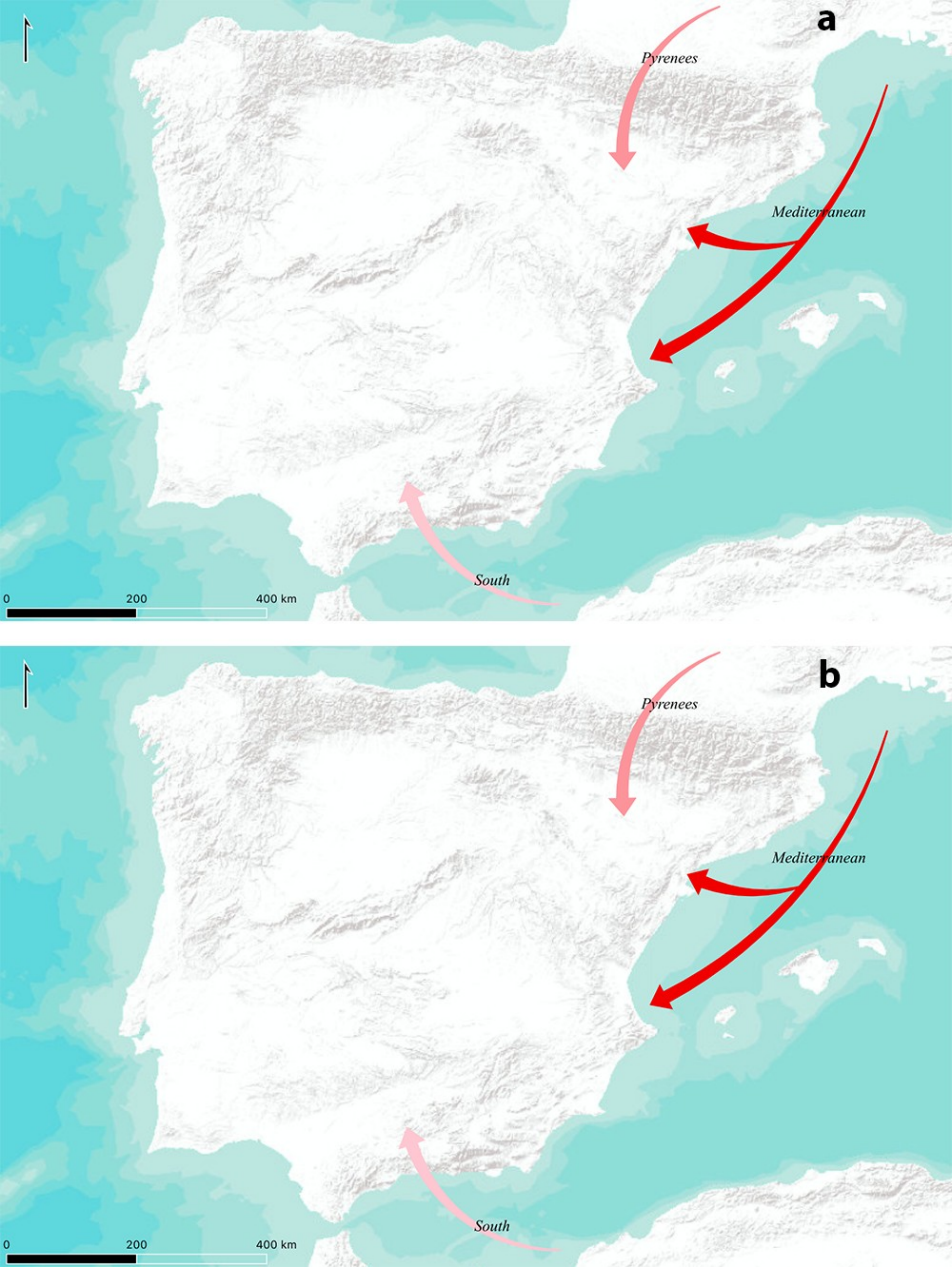
Probability for the proposed expansion routes considering a single origin. (A) relaxed kernel. (B) strict kernel. Stronger colour indicates higher probability. Maps modified from ESRI World Terrain Base Map.

As for the possible expansion routes, we have considered three main possibilities, all of them with support in archaeological literature to a lesser or higher extent. First, we have considered a *leapfrog* expansion based on maritime colonization through the Northern Mediterranean [10, 23, 26, 60]; second, we have also taken into account a possible way of entrance through the Central-Western Pyrenees [26, 31] and third, we include a possible South Mediterranean route, despite, or maybe because of the current archaeological debate regarding this possibility [10, 13, 107]. Again, if we consider the aggregated probabilities, we obtain a probability of 56.77% for the Mediterranean route, 25.52% for the Pyrenees and 17.71% for the Southern route if we consider the restrictive kernel, while the probabilities for the relaxed kernel would be 57.29%, 25% and 17.71% respectively (Fig 6).

### Double-origin

We have also considered the possibility that the arrival of the first farmers to the Iberian Peninsula occurred following two different paths of entrance and, thus, two possible origins. Again, the posterior distributions of the parameter *r* follow a similar behaviour to the single-origin option. In order to consider the aggregated probabilities, and because the resulting similarity is affected by the interaction of *o*_1_ and *o*_2_, we have decided to attribute the paired origins of each simulation to the different regions/routes. Thus, we consider all possible pairs of origins, including the possibility of a repeated origin; that is, two starting points chosen in the same region.

**Fig 7:**
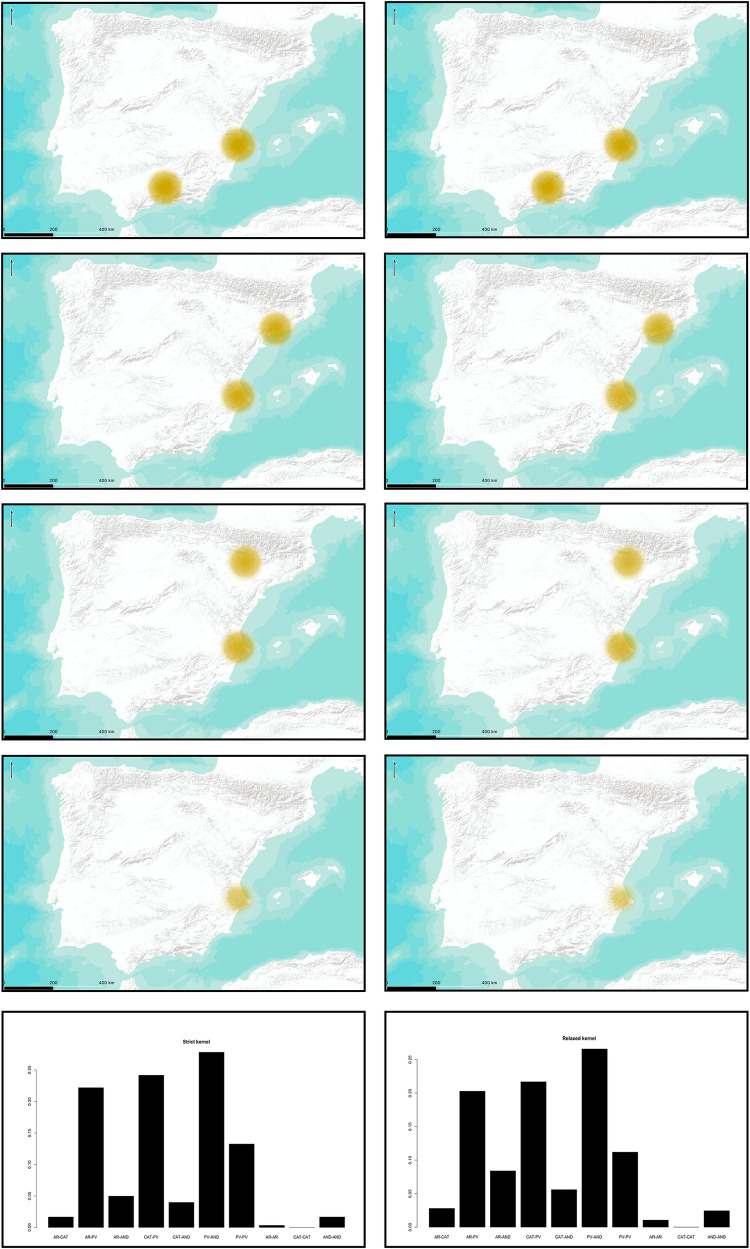
Probability for the proposed origin areas considering two origins. Higher intensity and size of circles indicates higher probability. Left column represents relaxed kernels; right column strict kernels. From left to right, barplot abbreviations stand for: AR-CAT = Ebro-Northeast, AR-PV = Ebro-Xúquer, AR-AND = Ebro-Guadalquivir, CAT-PV = Northeast-Xúquer, CAT-AND = Northeast-Guadalquivir, PV-AND = Xúquer, Guadalquivir, PV-PV, AR-AR, CAT-CAT, AND-AND. Maps modified from ESRI World Terrain Base Map.

**Fig 8:**
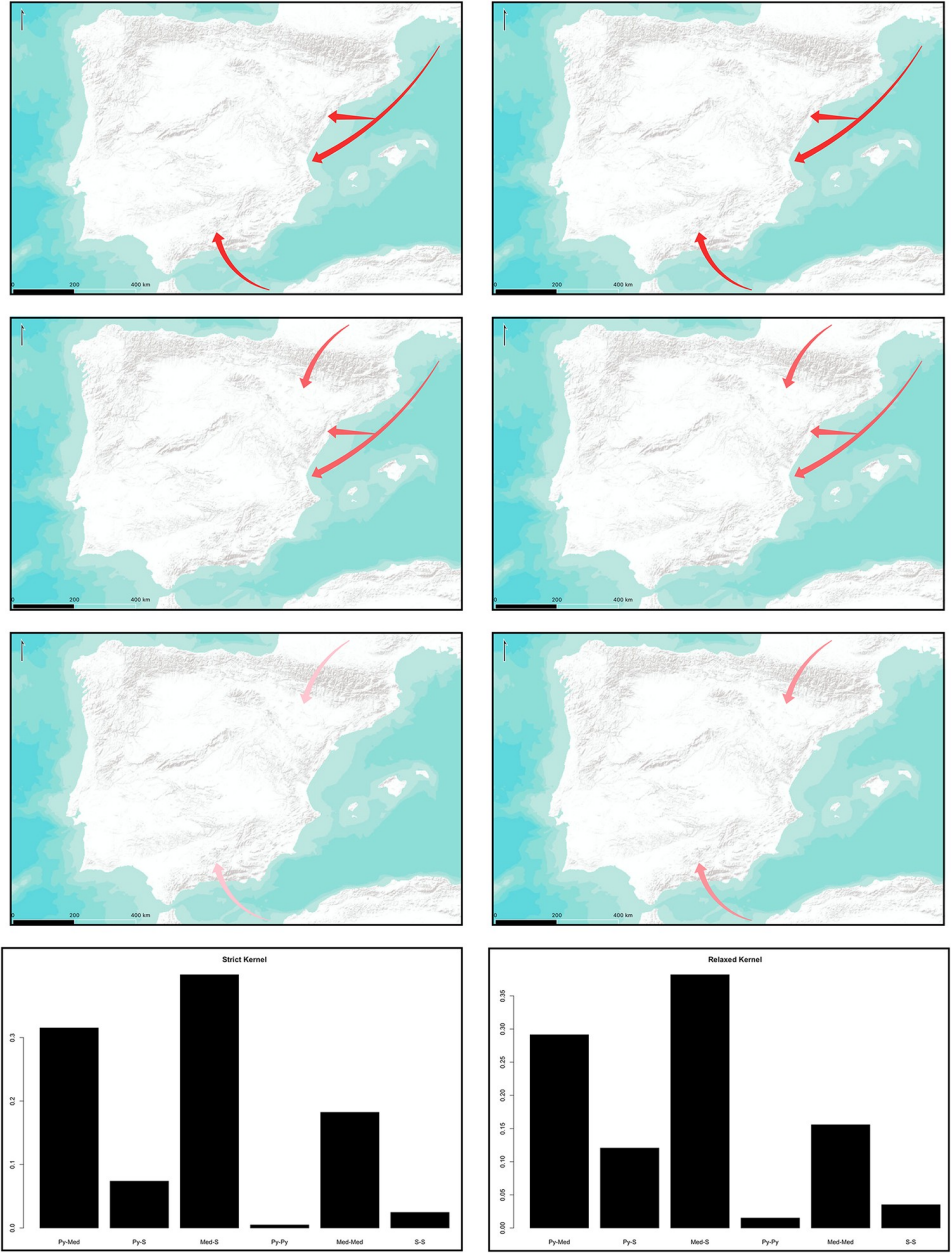
Probability for the proposed expansion routes expansion routes considering two origins. Stronger colour indicates higher probability. Left column represents relaxed kernels; right column strict kernels. From left to right, barplot abbreviations stand for: Py-Med = Inner Pyrenees-Northern Mediterranean, Py-S = Inner Pyrenees-Southern Mediterranean, Med-S = Northern Mediterranean-Southern Mediterranean, Py-Py = Pyrenees-Pyrenees, Med-Med = Northern Mediterranean-Northern Mediterrnean, S-S = Southern Mediterranean-Southern-Mediterranean. Maps modified from ESRI World Terrain Base Map.

As we can see in Fig 7, considering a possible double origin of the Neolithization process, the combined nuclei of the Valencian and the Andalusian shores seem to be the most likely regardless of *r* being strict or relaxed, with a 27.81% in the first case and a 26.57% in the second one. The inclusion of the Valencian Country into the possible origins increases the probability for each case, including a possible double origin in a single zone. This is in the line of the results proposed for a single origin. If we consider, as in the previous section, possible expansion routes, then again, the Northern and Southern maritime routes seem to hold the highest probability with 39.75% in the first case and 38.19% in the second case (Fig 8).

## Discussion

Once the terms of the results in regards with their statistical meaning have been explained we must highlight two interesting points, a) the existence of signals of cultural variability in stylistic traits of geometric projectiles according to geographical distance and b) the significant outputs considering specific points of origin. At this point, the hypotheses tested are relevant in the framework of recent archaeological literature regarding the Neolithization process at the Western Mediterranean although with the novelty of a new methodological approach based on crossing cultural variability data with chronological and spatial information. As we can see, three main routes, the northern Mediterranean, the Pyrenees and the Southern route (considering a possible dispersal from the Northern African shores) have been explored. Although the importance of sea travelling for the expansion of the Neolithic in the Mediterranean has been widely accepted by the archaeological literature [10, 12, 26, 29, 60, 108–111], some questions remain open, mainly affecting possible expansion routes and timing. While some authors consider the existence of a Southern Mediterranean route of expansion for the Neolithic spread in the Iberian Peninsula, either based on material culture (similarity in lithics and pottery) [107] or 14C dating [13], others reject this possible expansion route, based on the problematic sequences of Northern African sites, arguing that the Neolithic expansion would still follow the Northern Mediterranean route, and would expand from the Iberian Peninsula to Northern Africa [10].

In this matter, and following the results of this study, we have considered different possibilities for the expansion of the Neolithic in the Iberian Peninsula, not always necessarily exclusive of one another. In order to do so, we have combined our current information in 14C dating and material culture, focusing on geometric microliths. Our analyses point at the Northern Mediterranean as the most probable candidate for the Neolithic expansion, which also agrees with most of archaeological literature [109]. The Northern Mediterranean route is included in all of our most probable results. This also finds agreement with current archaeological knowledge, attributing the oldest *undiscussed* Neolithic dates to sites in this area, such as Mas d’Is [27] or Guixeres [30]. As for the other two routes considered, the inner Pyrenees route has been taken into account in the past, due to the old dates in Cueva de Chaves [26, 31]. However, this route has not found support in our statistical analysis. If we consider carefully the data for this zone with presence of Early Neolithic geometric microliths, there are only two sites, even considering the upper layers of Valmayor XI as Neolithic, despite the reserves posed by their main excavators [32]. Indeed, this finds agreement with the archaeological record found at the region where, although considerable survey efforts and excavations have been made [112], sites with *old* Neolithic dates seem to be scarce as of now.

Perhaps the case of the Southern route is the most complicated one. In our analysis, the probability of a second Neolithic origin point in the South of the Iberian Peninsula, which could imply a South-North Mediterranean advance from Northern Africa, is relatively high. João Zilhão [10] has strongly discussed against this possibility based mainly on: (1) because Early Neolithic navigation was based on cabotage and there is no material connection between Sicily and Tunisia, the step from Italy-Africa, necessary for the spread, could have not been made — although Freund and Batist [113], based on a network study of the distribution of obsidian in the Western Mediterranean, suggest that Early Neolithic seafaring would permit covering open-sea distances of ~200 Km; (2) if we do not consider sites with stratigraphic problems and long-live samples, the first Neolithic in the Maghreb dates to ~7350 cal BP, thus later than it has been recognised for the Iberian Peninsula and (3) unlike other Western Mediterranean areas, *cardial* culture appears *ex novo* in Northern Africa, without previous traditions.

**Fig 9:**
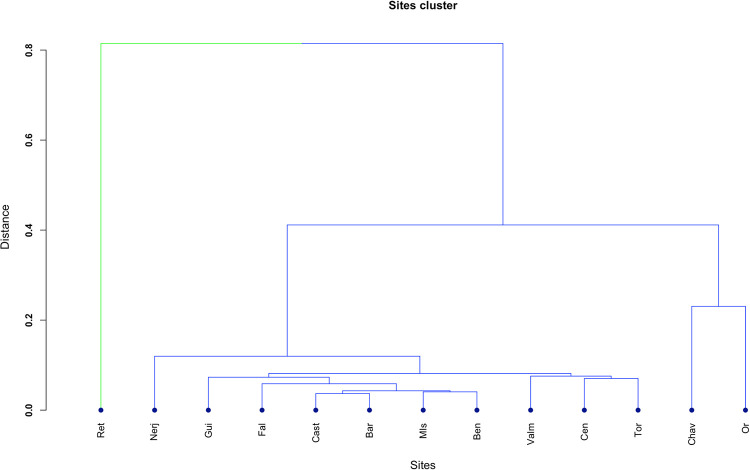
Sites hierarchical cluster on the basis of the similarity of their geometric microliths.

These arguments are consistent at the current state of research and, yet, the possibility of a Southern origin is still present in our results. After examining our data, we must consider the fact that we have included the site of Retamar in the analysis. This site was considered to contain Neolithic levels in its first interpretation [43]. However, more recent research has pointed out the possibility that its Neolithic levels are actually a palimpsest containing different chronologies [60]. In our study of the geometric microliths of the site, indeed some of the ones present in the Neolithic levels would actually show traits closer to Mesolithic technology and style (excess of concavity of their sides, consistent abrupt retouch, etc.). As a matter of fact, the site groups out of the rest of the sites when trying to hierarchically cluster them (Fig 9). We have decided to keep the site in the study because (1) the Neolithic *is* present at the site and (2) it does present old dates within the Neolithic range. Whether this is representing higher levels of horizontal transmission than expected, even for previous chronologies; it does actually represent a point of Neolithic origin; or is simply a bias product due to the admixture produced in the site will be considered in future work.

In any case, we must also consider the fact that having one possible origin in the Southern part of the Iberian Peninsula does not necessarily imply a South Mediterranean expansion route. If we consider an average of at least 300 km per generation expansion rate, being each generation 32 years [12], then the first Neolithic groups could have reached the shores from Catalonia to Málaga within a maximum of two-three generations; which could effectively turn the origin points as synchronic in our analysis. Indeed, if we consider again the individual site analysis, Nerja, and not Retamar (although, admittedly, there are very similar probabilities for every Southern site), appears as the most probable origin in the South, despite the fact that the oldest date could correspond to the latter.

Finally, we must take into account that, despite the fact that the origin probabilities for each area have been weighted to the number of sites per zone, there is indeed a potential effect in the analysis due to the number of sites present in each region. However, the number of sites per region is also part of the Neolithization process, even more so when considering that, for all of these areas there has been active archaeological research during the last years [114], thus the number of sites should not be considered as a potential bias, but rather as an increase in the information at our disposal.

In any case, this study seems to state clearly one possible expansion route following the Northern Mediterranean, in correspondence with current archaeological knowledge. For the Southern Mediterranean possibility, although it could be considered for future studies, there are currently other elements which must be disentangled in order to understand it better and, ultimately, the only answer to this could come from an increase on the well-stratified archaeological record from the Northern African shores.

## Conclusion

Cultural diversity in the Neolithic spread along the Western Mediterranean has been approached extensively considering different scales of the analysis from a geographical and/or a diachronic point of view. The first Neolithic package includes ceramic pots, new lithic knapped tools or polished stone among other cultural productions. In a general view, the early Neolithic groups have been characterized from pottery remains in a tradition conformed by the impressed pottery ware. As we have noted, recent archaeological discoveries and renewed radiocarbon records have revealed the oldest advance of pioneer *impressa* groups from the beginning of the VIII millennium cal BP. The discoveries in the Languedoc area (South of France) [29, 115] and its recognition in the Mediterranean Iberia (Barranquet site) highlight the complexity of the Neolithic dispersal process regarding different times and trajectories. As for the geometric projectiles the signals found through the present approach would account for geographic cultural diversity towards the far western Mediterranean, and a Neolithic dispersal pattern following the Mediterranean northern route.

In any case, if we were to focus on the possible reasons accounting for this variability in evolutionary terms, some questions emerge. Indeed, the closeness among the current radiocarbon dates raises some issues. Namely, the speed of the expansion and the proposed leapfrog movement [12, 115] would suggest a great similarity in stylistic traits. Isolation by distance could be one good candidate as a mechanism producing cultural diversity in a lower density population scenario [17, 53]. However, if that were the case, we would expect to see higher time differences on the arrival of the first Neolithic groups at the different regions, since the diachronic component is necessary in order to produce drifts leading to cultural dissimilarity. This is not the case for the radiocarbon record used here. At this point we must remark several features that can shed some light on this matter and undoubtedly merit more attention:

1. The radiocarbon record, although obviously related, does not seem in full accordance with the Neolithic origins modelled here. Indeed, we should probably consider it as rather the consolidation of the farmer groups in some regions. If this were the case, the expectancy would be to find older Neolithic radiocarbon dates at some specific locations, as the discovery of the early *impressa* ware to the Xúquer region would suggest. If we assumed an older Neolithic arrival, not recorded as of now, then we could consider diachronic drifts leading to cultural dissimilarity.
2. After the initial Neolithic demic contribution, the interaction between Mesolithic and Neolithic groups could be in some way significant considering the weight of cultural transfers and borrows in a shared and common lithic pool by both. To confirm this point, it would be necessary to add some new parameters and methods able to explore diversity taking into account different regional outputs [111].
3. Obviously, the nature and size of the sample analysed must be improved and increased, while it is also necessary to test the method including other cultural items. In fact, some works designed to explore cultural diversity in a diachronic view, and focussing on pottery and ornament have revealed different mechanisms of cultural transmission [17, 18, 53].

Summarizing, we have designed this work as an analytical procedure to look into cultural patterns for the agriculture spread in Iberia. The combined approach of cultural and geo-chronological similarity analysis, aided with the computational power of the ABC-SMC methods opens a new window to simulate and model crucial questions accounting for cultural diversity in a human dispersal scenario. Improving the method, exploring new parameters, and extending the spatial and temporal contexts constitute part of the challenges to build new evolutionary histories in regards with the Neolithic transition in the western Mediterranean.

## Supporting information

S1 File. Methods and data Detailed discussion of the methods and data used. (DOCX)

S2 File. Reproducibility Includes all data and scripts necessary for full reproducibility of the paper. It includes a readme file with further indications.
(ZIP)
