# Supplementary material for: The spread of agriculture in Iberia through Approximate Bayesian Computation and Neolithic projectile tools
Source: PLoS One. 2021 Dec 28;16(12):e0261813. doi: 10.1371/journal.pone.0261813 (PMC8714124; doi:10.1371/journal.pone.0261813)
Supplement: S1 File. Methods and data — (DOCX) [file pone.0261813.s001.docx]

**METHODS AND DATA**

**1. Sites**

Cueva de Chaves: Cueva de Chaves is placed at the Barranco de Solencio, in the Guara Mountain range, the southern and lowest sector of the High Aragon in the Pre-Pyrenees, 60 km away from the Pyrenees and at 663 masl [1]. Initial systematic excavations happened first in 1974/75 and later in 1984 and 1990. The denomination of the stratigraphic sequence was set in the latest campaigns, when a Palaeolithic phase and two Neolithic episodes were recognized, Level 1b attached to a cardial moment and Level 1a, occupied some time during the recent cardial [2]. In this case, the materials used in the study belong to Level 1b dated on 6580 ± 35, (GrA38022) from *ovis aries*, and including material recovered between 1984 and 1990 exclusively [2]. For the present work, Chaves’ assemblage is one of the most numerous, with a total of 42 pieces included ([2] pages 138, 139, 145 and 151).

Valmayor: The site of Valmayor is located in a 120 masl shelter rock, strategically positioned between the Ebro and Matarraña valleys, in a sandstones and water catchment area. Although the site was known in the 1980s due to rock art studies [3], the development of the archaeological sequence is due to the systematic excavations undertaken in 2011 [4]. Latest studies have noted three occupation phases occurring in different moments during the VIII millennium cal BP, Valmayor XI-I, XI-II and XI-III, with a phase of population gap between the last ones. This study includes the geometrics located in Valmayor XI-II ([4] page 53) with a date of 6570 ± 30 (Beta341168) on animal bone. Valmayor XI-II has initially been proposed as a Mesolithic site with influence of the nearby Neolithic settlements [4]. However, recent reinterpretations seem to point to a fully configured Neolithic settlement [5].

Can Sadurní: The site is a 200 m2 long cave located near a wide terrace in the middle of the Massís del Garraf, controlling the mountain valley at 450 masl [6]. Human occupations in the cave are well-known since the 1980s [7–10], when a wide stratigraphy ranging from postcardial to medieval chronologies was noted [11]. Interventions continued from 1992, including different diggings inside the cavity, an open excavation area and a trench [6,11]. The trench area provides the archaeological material used in this study, one geometric microlithic located in the layer 18, a cardial sepulchral level ([6] page 630). However, because this geometric did not meet our reliability criteria (the piece had large broken missing parts), the site has been discarded from the study.

Guixeres: Guixeres is an open-air site, located on the top of the Vilobí cretaceous outcrop, at 330 masl [12]. The systematics works in the site, which first lasted from 1974 to 1984, and were retaken in 2015, exposed a stratigraphic sequence with three occupational events, Phase A or Ancient Cardial Neolithic level, Phase B or Ancient Epicardial Neolithic level and Phase C or Cardial Post-Neolithic level [13,12,14]. In the ancient one, Ancient Cardial Neolithic, two habitation areas were documented, Ámbito 1 and Ámbito 2, with numerous negative structures such as hole posts and silos ([14] page 37). We use the site’s oldest date on *ovis aries*, 6655 ± 45 (OxA26068), which happens to be one of the oldest ones for the Iberian Peninsula. Five geometric microliths from the Ancient Cardial Level have been used for this study; four of them retrieved from Mestres [12] and the last one from Gibaja et al. [14].

Barranquet: Located in Oliva (València), Barranquet is an open-air site formed by paleochannels in the Ancient Neolithic period, at 3 km from the coast [36]. The archaeological work was motivated by the execution of an urban project and exposed four occupational levels, including contemporary buildings. The Neolithic level occupied a 305 m^2^ extension area, where anthropic structures were not identified, probably due to post-depositional processes. For this site, *impressa* decoration pottery has been discovered, and it presents a 14C dating of 6510 ± 50 BP (Beta221431) on *ovis aries*, and 2 geometric microliths for the Ancient Neolithic level (UE79).

Cova de l’Or: The cave is located on the eastern side of the Benicadell mountain range, 650 masl (Alacant). The first excavations recovered most of the material from the site and took place in the middle of the 20^th^ century by Vicent Pascual. Later excavations were developed by Martí during the 80s of the same century. The archaeological sequence of Cova de l’Or includes different Neolithic phases, namely, Early Cardial Neolithic (Neolithic IA) with cardial ware, and Early Cardial/Epicardial Neolithic (Neolithic IB) among others recent episodes. In order to retrieve most of the material, we have followed the periodization proposed by Juan-Cabanilles [33], considering only his Neolithic IA and adding the oldest levels of the most recent excavations ([34,35] but not fully published). This work includes 24 geometrics belonging to this site/period, dated to 6510 ± 160 (KN51) on short-live sample.

Benàmer: Benàmer is an open-air site located in Muro d’Alcoi (Alacant), at the Serpis valley, 20 meters above the riverbed. The archaeological intervention occurs in 2008 and 2009 due to the construction of a motorway [32]. The sequence presents different and discontinuous occupational phases from the IX millennium cal BP to the IV-III centuries BC, including Mesolithic and Neolithic levels with a gap of ~900 14C years. Particularly, the Ancient Neolithic is found in level 2, presenting combustion structures and consumption and production areas with a 14C of 6575 ± 50 BP (CNA539) on pollen and 2 geometric microliths belonging to this period.

Cova de les Cendres: This site, a large and unclosed cavity located in Teulada-Moraira (Alacant), corresponds to an ancient karst level developed in the stretch of sea cliffs between Cap de la Nau and Puntal de Moraira. The archaeological works affected ~50 m^2^ of the cavity, and provided a considerable sequence, presenting Paleolithic, Neolithic and Bronze age chronologies. Its Neolithic levels were excavated during the 80s and 90s of the 20^th^ century [37], and have been reopened again since 2018. We have used 5 geometric microliths, found at the base of the Neolithic sequence, dated 6510 ± 40 BP (Beta239377) on domestic animal.

Mas d’Is: This open-air site, placed in an old glacis between two ravines in Penàguila (Alacant), has known a great number of campaigns from the middle 90s of the 20^th^ century. Different domestic structures were found at the centre of the site, among them some identified huts. The Ancient Neolithic presents a date of 6600 ± 50 BP (Beta162092) on *hordeum sp*. [39] and *Impressa* decorated ware has also been found in this site. Two geometrics belong to this level.

Abric de la Falguera: Abric de la Falguera (Alcoi, Alacant) is placed in the Barranc de les Coves. The site was first excavated during the early 80s of the 20^th^ century, and then excavated again in the period 1998-2001 [38]. Latest works established the archaeological sequence base. A total of eight cultural phases have been identified, with a Mesolithic one on the base and a contemporary stage on the top. The Ancient Neolithic is found in the level VII of the sequence, it has been dated at 6510 ± 80 BP (Beta142289) on *triticum monococcum*, and we have retrieved 3 geometrics.

Los Castillejos: The site of Castillejos, another open-air one, is placed inside Las Peñas de los Gitanos, an archaeological area surrounded by megalithic assemblages and different caves studied since the beginning of the 20th century [15,16]. The interventions developed in 1971 and 1974 completed and defined the stratigraphical sequence of the site [17]. This information was enlarged from succeeding campaigns in 1991 and 1994 [18–20]. The sequence suggests a discontinued habitat from the VIII millennium cal BP, and we have used the date from 6310 ± 45 (Ua36215) for this article [15]. A total of two geometric microliths has been selected.

Cueva del Toro: Toro cave is located at 1190 masl in El Torcal de Antequera, a karst mountain range which restricts the province of Málaga to the North. Five systematic excavation campaigns (1977, 1980, 1981, 1985 and 1983) have evidenced a stratigraphic sequence which reflects human occupation from the middle of the VIII millennium to the beginning of the IV millennium cal BP [21]. Phase IV, dated to 6320 ± 70 cal BP (GrN15443), corresponds to an Ancient Neolithic habitation, where the exploitation and the posterior treatment of meat products seems to be the main activity [21,22], 4 geometric microliths have been selected in the study ([23] page 40).

Cueva de Nerja: This site is placed in a large cave located 158 masl South from the Almijara Mountain range, 1000 m away from the coast. According to the material record [27], the external rooms of the cavity have been occupied since about 25000 until 4000 BP, including Upper Pleistocene and Chalcolithic phases. Archaeological interventions in the cavity have been diverse and dilated in time, including general parietal prospections and systematic diggings in the externals rooms of Mina, Vestíbulo and Torca [28,29]. The materials used in this study, 10 geometric microliths, come from different areas, Vestíbulo (NV) and Mina (NM), and levels, NV2, NV3 and NM9, NM10, recovered during Jordá’s interventions (1979-1987), using the date of 6590 ± 40 from a *ovis aries* bone (Beta131577) [30,31].

El Retamar: The open-air site of Retamar is placed in a coastal environment, in which is currently a bay formed after the occupation of the site. Excavations and material studies were directed by José Ramos Muñoz. Retamar has been interpreted as a settlement occupied by communities with a livestock base completed by fishing and shellfish collecting activities ([24]). The published radiocarbon dates from the malacological record seem to refer several episodes including early Neolithic occupations. At this point we consider the date 6780 ± 80 BP (Beta90122). In Retamar, the lithic record recalls to some assemblages found in the Epipaleolithic layers of nearby sites (Palmones, Nerja, Hoyo de la Mina), a fact which points to communities with Epipaleolithic traditions which included, according to the author, Neolithic production standards [25]. The record of Retamar has been extensively published [26,25], including a monograph [25]. The last one offers a rich graphic material from which the necessary data for the study has been extracted ([25] pages 289-337). All in all, we have used 58 geometric microliths from Retamar.

**2. Geometric projectiles**

2.1 Collecting data

Most of the data has been gathered automatically using the R package GeomeasuRe [40,41]. GeomeasuRe is a package able to capture different size measures directly from a vectorized picture. In this sense, for some sites we have inspected the collections personally (Benàmer, Cova de l’Or, Barranquet, Cova de les Cendres, Abric de la Falguera, Mas d’Is) while for some others vectorizations have been developed from the published images (Cueva de Chaves, Valmayor, Can Sadurní, Guixeres, Los Castillejos, Cueva del Toro, El Retamar, Cueva de Nerja).

Although the exact functioning of the package is developed in the aforementioned references, some comments might be due. To collect the data using this system, the geometric microlith is deployed over a 10 cm long grid (for publication retrieved data, the geometric must be scaled previous to the vectorization), after which the image is vectorized and produced at natural size as a .*shp* spatial object. Then, the image is imported to R [42], where the measures are taken. Asides from standard measures, such as general length and width, area, angles, reliability and symmetry, the key aspect of the package is the L-lines. The L-lines are a series of lines crossing the geometric from the aforementioned grid with a separation of 1 mm. Each of these lines is measured as it crosses the piece, thus giving information of the size of the geometric at each millimetre, both length and width-wise (Figure 1).


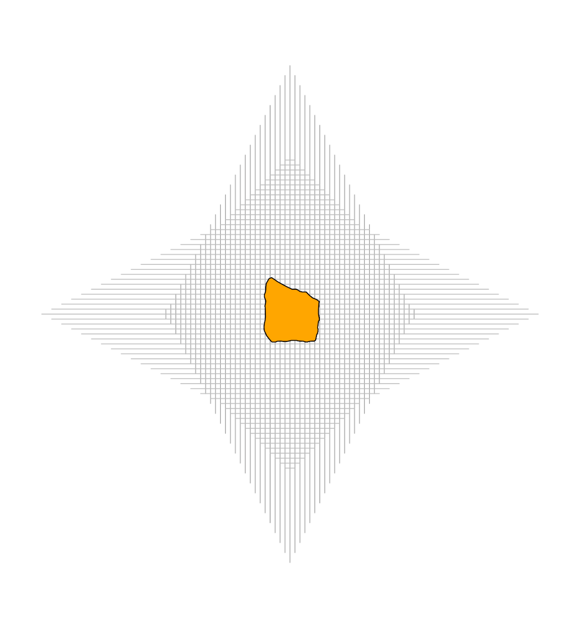

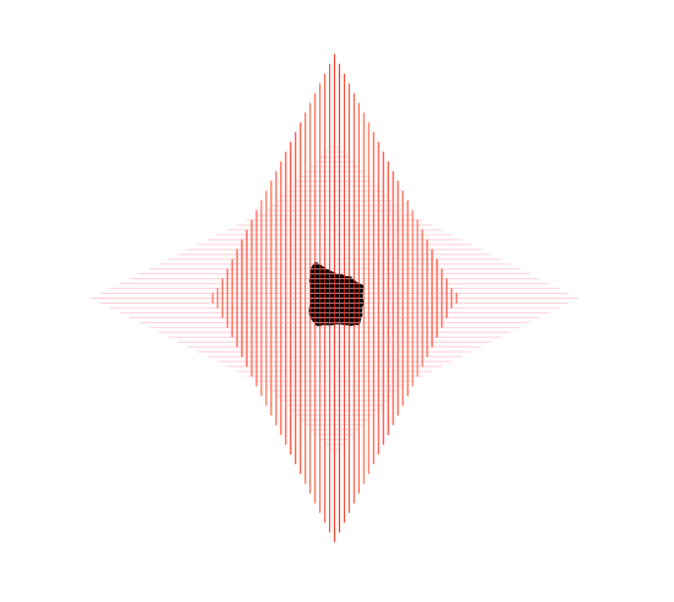


**Fig. 1. Geometrics over pre-defined grid.** The parts of the L-lines crossing the geometric are taken as the measure of the geometric at that specific point.

There are two ways of collecting the L-lines, the first one measures the whole length and the whole width of the geometric at each crossing L-line. This produces a total of 114 measures (51 for length and 63 for width). The second one, and the one used in this work, divides the geometric in halves, and then takes the measures separately. In this way we can be more accurate in capturing side difference, which is key in the problem approached here. More specifically, the geometric is divided in two sides; one proximal side and one distal side. Then the length distances are measured separately for the distal side and the proximal side. After this, the geometric is divided in other two halves, minor base half and major base half, and the width distances are measured separately for each. This means multiplying the measures, thus ending with a total of 233 measures. To this we must add the total length, total width, area and proximal and distal angles, which returns a total of 238 measures. However, it must be noted that not all of these measures will produce usable results. For example, as we have seen in the previous figure one, the geometric will return 0 in many of the L-lines, as it does not occupy the whole grid. How both these 0 values and the large number of variables are treated will be shown in the next paragraphs.

2.2 Reliability

The package also measures the reliability of the measures for the case of broken pieces by computing how much of the piece is missing based on the most probable shape of the complete piece, and penalising pieces with large missing parts. This is returned as a percentage value. For this article we have only considered pieces with a reliability index > 95%. Because of this restriction, we have had to discard the site of Can Sadurní, as it only contained one geometric (for the concerning chronologies) and it did not meet the reliability criterion. Furthermore, the GeomasuRe package also captures whether each L-line is complete for the non-complete accepted geometrics (this is $95\leq Rel\leq100$). The non-complete L-lines are given NA values and treated accordingly.

2.3 Scale and centring

Scaling and centring are usually advised when dealing with morphometric assessment. Here, we have performed both operations in order to eliminate possible problems due to different raw material sources. In this sense, one of the most common procedures is using the so-called Procrustes General Analysis (PGA) within the Geometric Morphometrics approach [43–45]. This method centres the samples and fits them all to the same size, so that measures are comparable (see [46] for a detailed description). It is very useful when dealing with somehow similar shapes, for which it was originally designed. However, the geometric microliths can be very variable in terms of length, width, ratio and general morphometry. Therefore, the rationale of factor scaling can result in samples where the length is similar but the width is not, and vice versa. Because of this, we have decided to scale each side separately. In this way, we ensure that almost the same measures will be taken for each geometric, and we can capture the morphometry of the sides more accurately. Finally, the geometrics have all been centred and orientated with the minor base (vertex for the triangles and arch for the segments) to the right. Although we acknowledge that this contravenes the standard orientation procedure [47], we believe that it produces a better behaviour for automatised analyses such as this one.

2.4 Factor variables

We have also considered categoric variables, which cannot be automatically extracted. In this sense, we have considered, for each geometric the direction and the mode of the retouch for each side. These variables have been observed personally, either directly or from published sources, for each geometric.

2.5 PCA

In order to deal with this large number of variables, we have performed a Principal Component Analysis (PCA), a very well-known technique for dimensionality reduction, on the continuous variables. However, previous to this, we have filtered the non-meaningful data. To do this, we have removed all columns-variables that did not present measures > 0 in at least more than 30 observations, the minimum number advised when a distribution can be considered asymptotically normal. If the remaining columns presented NA values at any observation (due to the L-line being incomplete there), we have substituted that value by the mean of the column. Finally, we have removed the total length/width variables, as well as the proximal and distal angles measurement, as we consider that they are already captured by the L-lines. This has already meant a substantial dimensional reduction, where we consider $d=117$ for the following PCA.

For the performance of the PCA, we have first computed the Mahalanobis distance in search for possible group outliers (Figure 2), and found no significant outliers. Furthermore, and after the observation of the standard deviation for each variable, we have decided to use a correlation matrix, also considering that variables are measured in similar magnitudes.


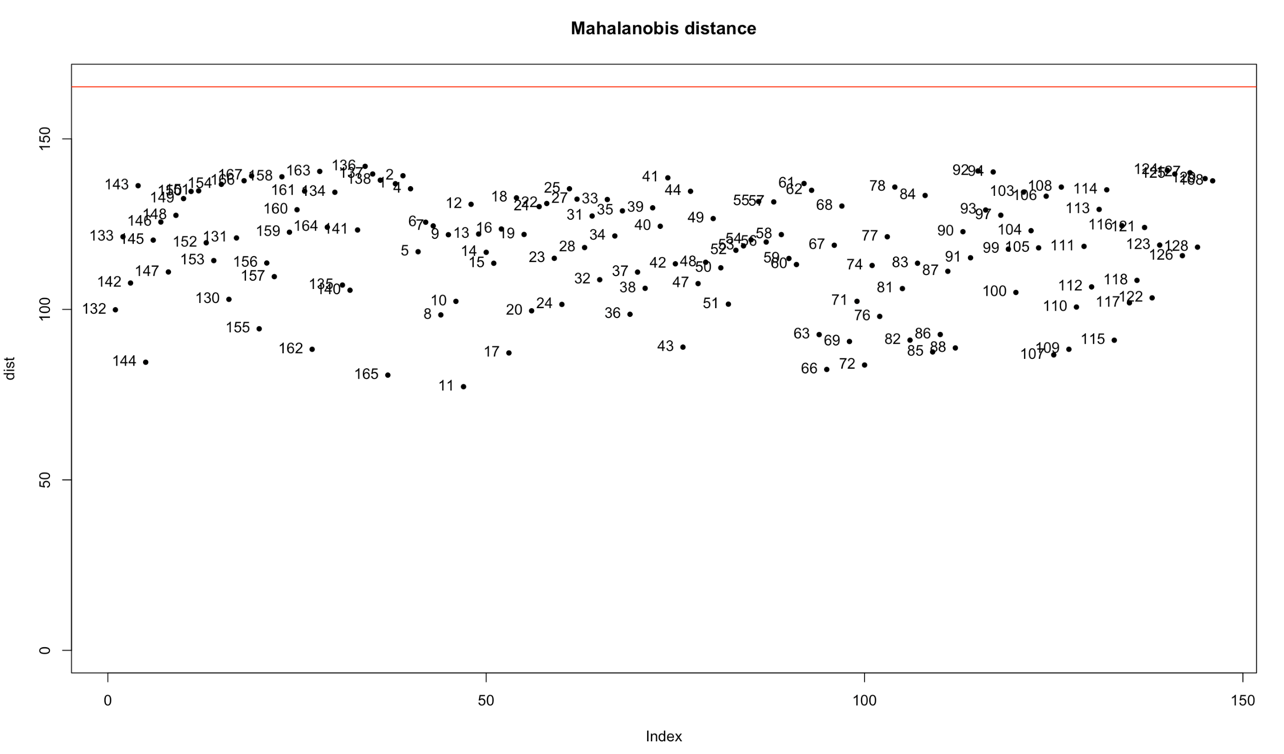


***Figure 2. Mahalanobis distance.*** *It shows no group outliers*.

After performing the PCA, we have decided to keep the first 15 components, which justify a 90% (0.897) of the variance (Figure 3). Although we acknowledge that setting a limit of 90% for the variance is somehow arbitrary, and that the first and the second components justify most of the variance by themselves (56.39%), we have preferred to keep a high value of morphometric justification, due to the aforementioned morphometric variability of the geometrics.


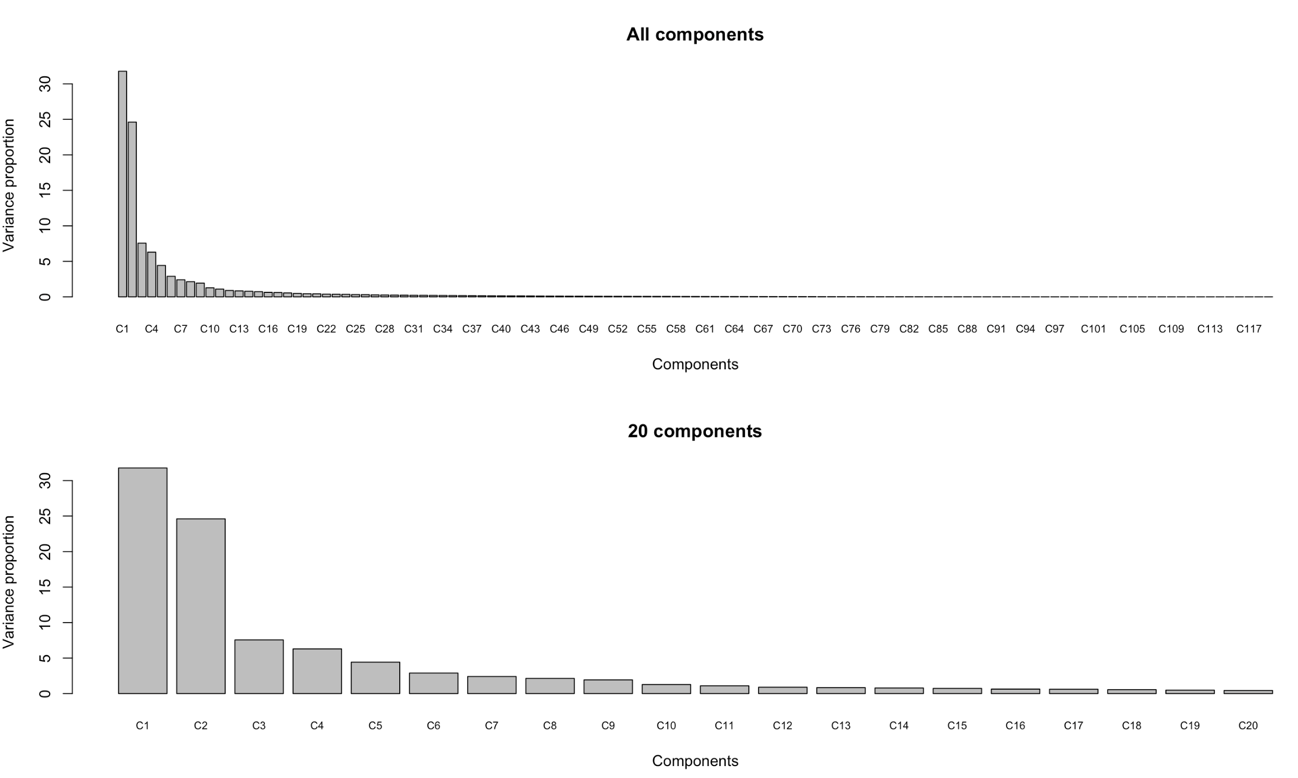


***Figure 3. Scree plot for the PCA*.**

2.6 Site data

However, our main analysis will be performed on a per-site basis. Therefore, in order to adapt the data for the statistical analysis, we have categorised each of the 15 components. We have assigned 5 categories for each component, this meaning that we have a 15 x 5 matrix with the PC components extracted from the L-lines for each site, where 15 is the respective PC and 5 is the categorisation of each PC. To this, we must add the retouch categoric variables. Finally, the per-site variables are obtained simply by counting how many of these traits are present per-site (see SO2).

**3. Statistical analysis**

3.1 Mantel tests

As specified in the main text we use partial Mantel tests to assess for spatial relationships. The overall idea is to create a distribution of significant Mantel’s *t* statistics. This distribution will be used as the observed statistic $S_{o}$ and will be compared to the simulated distributions, or simulated statistics $S_{s}$ through the SMC-ABC process (see next section).

Thus, we use three diversity matrices A, B and C.

*Matrix A*. This matrix accounts for cultural distance. We have created a distance matrix for the sites, using the Gower distance, well-suited to measure categoric variables.

*Matrix B*. The second matrix contains the geographic distance. It basically reflects the Euclidean geographic distance between each site.

*Matrix C*. Also using Euclidean distance, this matrix is based on the chronologies of each site. We have selected the oldest Neolithic BP date for each site, by sampling it from the probability distribution of the calibrated BP date, as explained in the main text.

Usually, for partial Mantel tests, the third matrix is used to control for the other two, and this matrix is held constant while the relationship between the other two is being determined [48]. However, we use a slightly different approach here. In creating the distribution of $S_{o}$, and because we cannot be sure that we have chosen the most probable calibrated date, due to the sampling process described in the main text, we create a first probability distribution with $n=1000$ observations. This solves two problems; first, since Mantel tests are based on permutations, the results are not exact, and this approach allows us to account for that; second, it also allows to prevent problems derived for bad choosing of the calibrated dates. It should also be noted that only significant Mantel *t* statistics are passed to the construction of the probability distribution, and that we have used Spearman correlation coefficient, more suited for non-parametric data.

It has been noted that Mantel tests are prone to type I errors in presence of spatial auto-correlation [49], and that partial Mantel tests are not always able to solve them [50]. Due to the fact that most of our variables were derived from a PCA (therefore orthogonal) analysis, this was unlikely. However, and because there have been steps after the PCA in the construction of variables, we have decided to perform the Moran’s I test for each variable, which proved either non-significant or not correlated, as expected (Figure 4 and table in SO2).


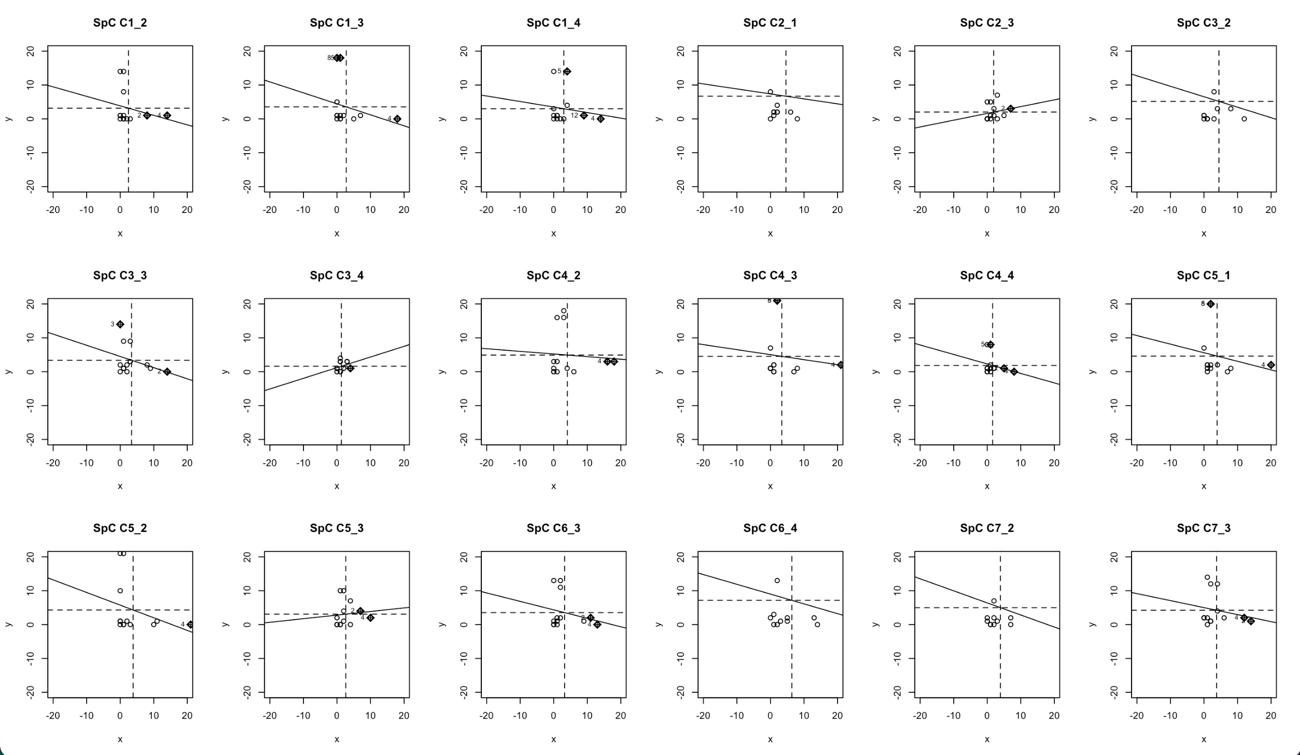


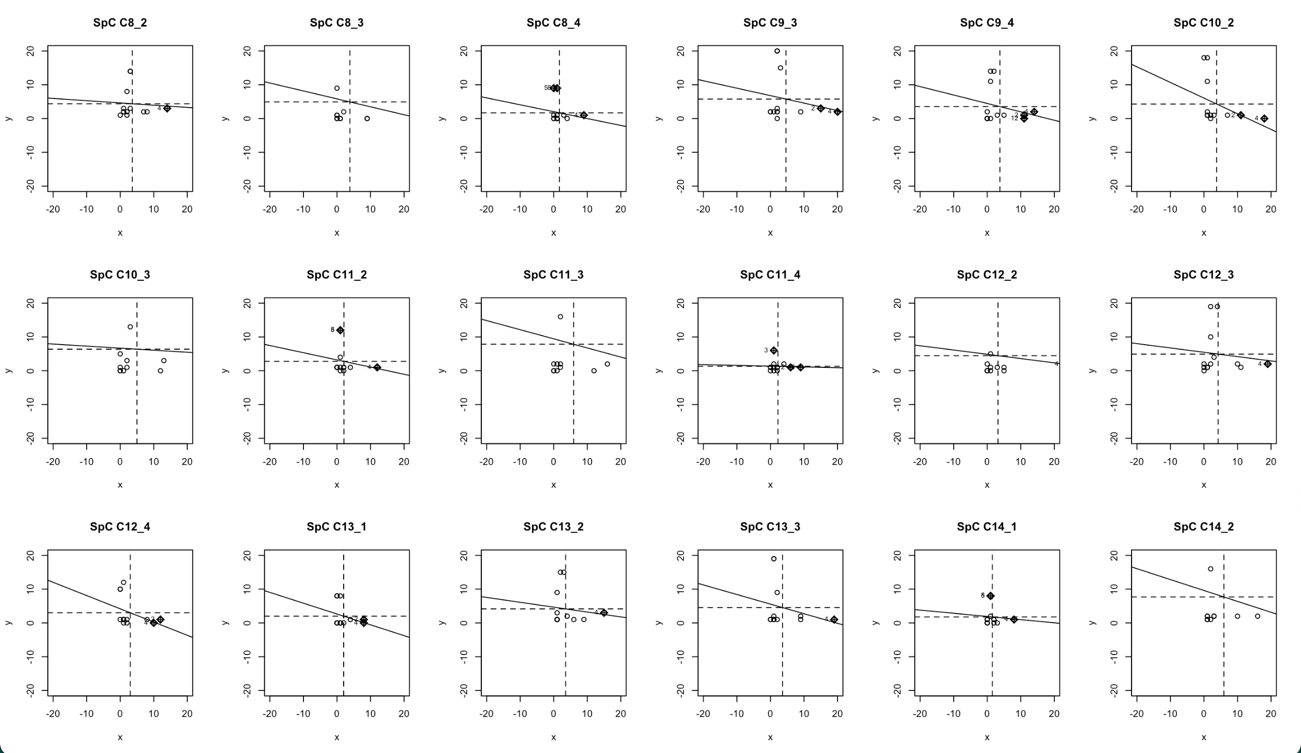


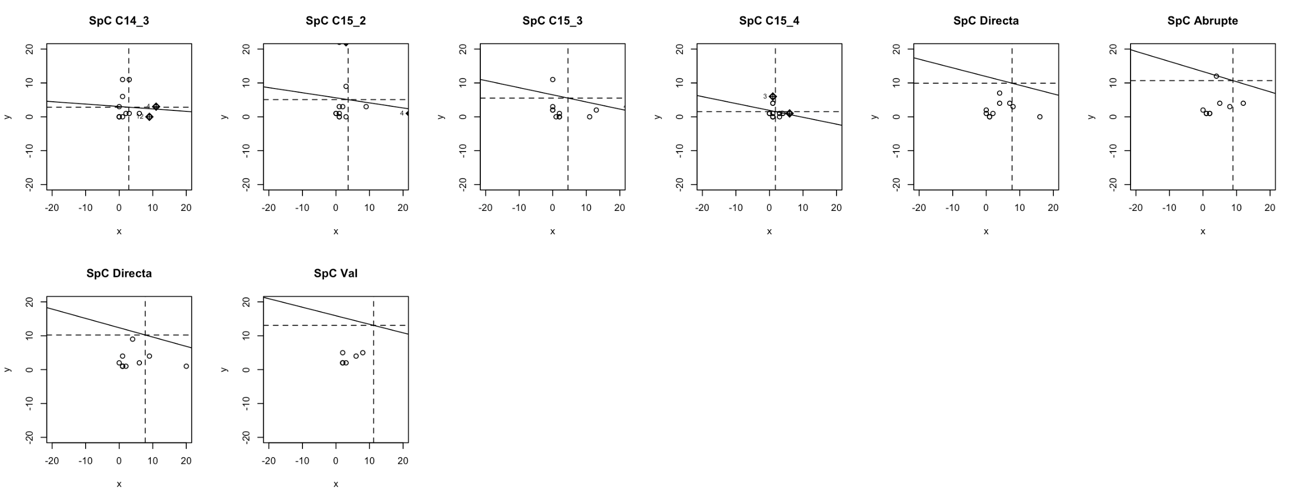


***Figure 4. Moran’s I results for each variable.*** *They confirm no spatial auto-correlation*.

Then, once we have the distribution of the Mantel’s *t* statistic, the idea is to compare that distribution to the possible distributions attending to different origin points, considering the 13 sites under study. The rationale is that for the simulated statistics $S_{s}$, since the cultural and geographic matrices A and B are equal both for $S_{o}$ and $S_{s}$, the changes in chronological matrix C will produce different Mantel’s *t* results. Thus, we perform a series of simulations following an expansion model with randomised origin points which proposes different chronological scenarios. These scenarios will also produce a simulated Mantel’s *t* distribution. The idea is to obtain the posterior distributions of the origin points proposed by the model.

3.2 Simulations

The key of the process is to simulate, and thus propose an alternative matrix C in order to compare different possible expansion routes to the known information, attending to the existent cultural similarity. In order to do so, we use a very simple expansion model,

$$y_{i}=t-\frac{o_{i}}{r}$$

Where *y* is the vector for the new dates per site, *t* is the calibrated date of the site randomly chosen as starting point, *o* is the vector of distances per site and *r* is the expansion rate, which follows a distribution $\sim U(1,5)$, in accordance with current literature [51]. For the sake of simplicity, we have decided to assume isotropy, and thus Euclidean distance. Although we are aware that anisotropy and difference in the velocity of expansion might have played a role in how culture is distributed, this is probably more detectable for smaller spaces, whereas for large geographic areas such as this one, the decay of cultural similarity can be considered isotropic [52]. Again, we have the problem of the calibration of the 14C dates. In order to select the starting date *t* we have followed the same procedure described in the main text, where the calibrated date is selected from the probability distribution of the calibrated BP date of the randomly chosen site. However, because simply reproducing the expansion on calibrated dates would not take into account the calibration error, we have back-calibrated and then calibrated back each date in order to reproduce that error, following the procedures described by Crema & Bevan [53].

Ultimately, from this equation we can obtain a new similarity matrix C_s_; from which we can perform a new partial Mantel test using the fix matrices A, B and the control matrix C_s_. Finally, iterating this process we will obtain the $S_{s}$ distribution.

We have also considered the possibility of two possible starting points for the Neolithic spread. In this sense, the model proposed is very similar, although considering two possible origins,

$$y_{i}=t_{1}-\frac{o_{i}}{r}$$

$$y_{i}=t_{2}-\frac{o_{i}}{r}$$

Where the date $y_{i}$ accepted for each site is the oldest one produced between the two origins $t_{1}$ and $t_{2}$. The rest of the process remains the same.

3.3 SMC-ABC approach

We have decided to use a Sequential Monte-Carlo – Approximate Bayesian Computation (SMC-ABC) approach, able to configure posterior distributions with more efficiency than the traditional rejection algorithm [54]. We have constructed four particles $\theta_{i}$, where the first particle from the rejection algorithm and next particles are constructed using the particle $\theta_{i-1}$ as prior. To construct the posterior, first the rejection algorithm is applied, where only simulations within an acceptance threshold based on pre-selected summary statistics are accepted. In our case, and because we are dealing with distributions, in the first case we only accept simulations falling within (Q_1_, Q_3_) of $S_{o}$. Then, as explained in the main text, this threshold must be reduced in an attempt to reduce the kurtosis of the posterior. Thus, for next particles the acceptance thresholds have been set to (P_30_, P_70_), (P_35_, P_65_) and (P_40_, P_60_), respectively. All particles are finally constituted by 1000 simulations. For the perturbation of the parameter *r*, we have chosen two different kernels; a more restrictive one, where $\sim U(\frac{r}{100}\times90,\frac{r}{100}\times110)$, and a more relaxed one, where $\sim U(r-1,r+1)$.

After this process is developed, we are interested in the parameter *o* (the origin point selected for each simulation), which is distributed as $o \sim Po(\lambda)$. The exact value of the parameter $\lambda$ of the posterior distribution is unknown, but it can be easily approached using a Poisson-Gamma distribution, where $Ga\left( \alpha,\beta\right)$ is the conjugate prior. Thus, considering the particle $\theta_{4}$ the posterior values of $\lambda$ distribute as,

$$\pi\left( \lambda| \mathbf{x} \right)\propto\lambda^{\sum x_{i}+\alpha-1}e^{-\lambda\left( n+\beta\right)}, \lambda>0$$

Which yields $E(\lambda)$ = 76.92 with 95% confidence interval between 72.23 and 81.76. However, in this case, we are not interested in $\lambda$, but in the mode of the distribution, where $P(Mo)\geq P(x_{i})$. The rationale is that the site that has been chosen as a starting point for similar significant Mantel’s *t* distributions has more chances of reflecting one of the starting points of the Neolithization process.

Finally, because we are aware of the strong random component of the model due to the randomization process for selecting the calibrated starting dates and in order to check for previous consistent results, we have performed a series of rejection algorithms with random seeds, under the rationale that all of them should produce somehow similar distributions. The results in figure 5 show that the model can approach correctly, and thus we have performed the rest of the simulation process.


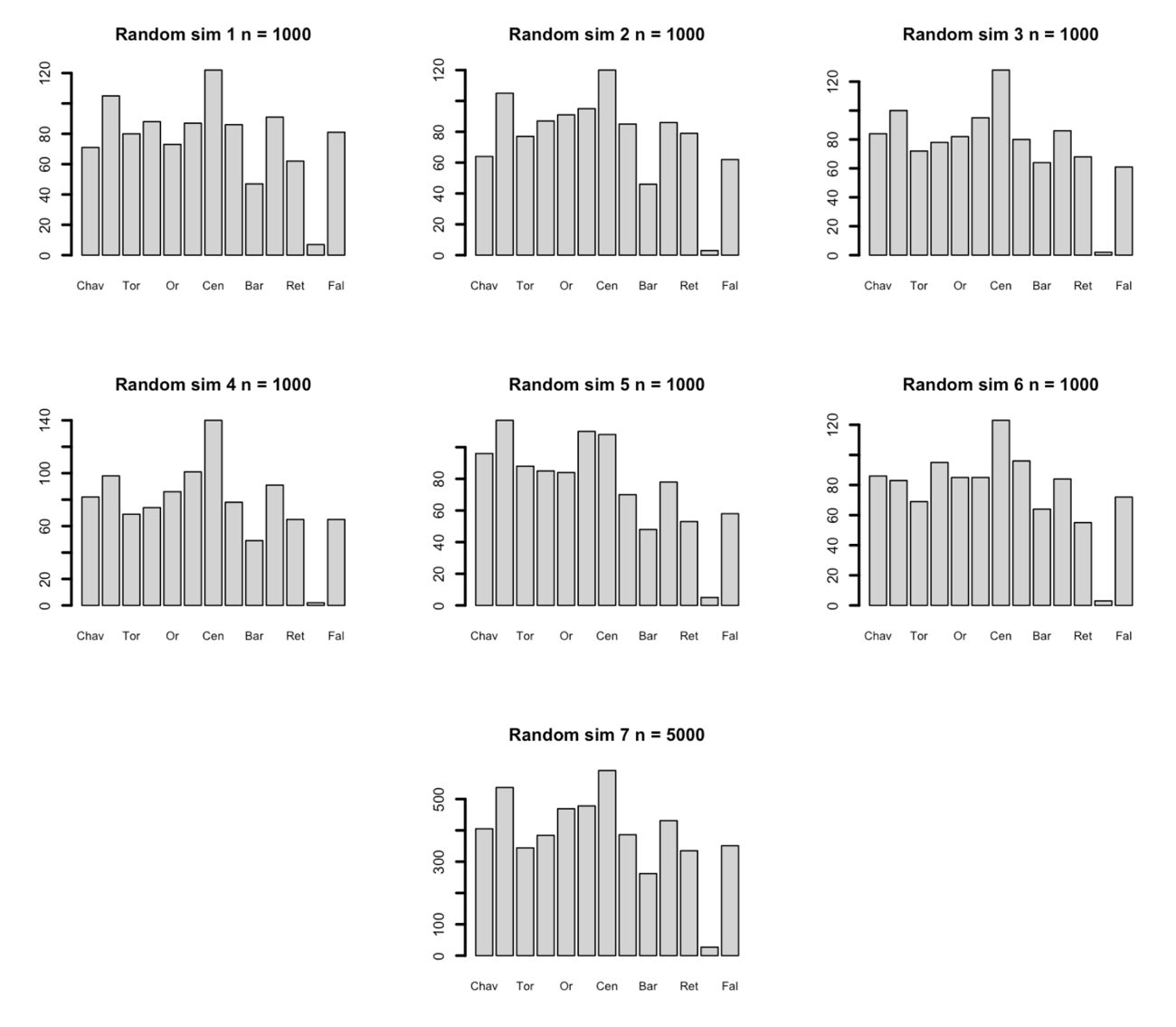


***Figure 5.*** ***Previous simulations with different random seeds.*** *They show the ability to converge to a good estimate of the posterior distribution.*

References

1. Baldellou Martínez V, Castán A. Excavaciones en la cueva de Chaves de Bastaras (Casbas-Huesca). Bolskan: Revista de arqueología del Instituto de Estudios Altoaragoneses. 1985;(1):9–38.

2. Cava Almuzara A. La industria lítica del neolítico en Chaves, Huesca. Saldvie: Estudios de prehistoria y arqueología. 2000;(1):77–164.

3. Royo Guillén JI. El abrigo con grabados rupestres esquemáticos de ‘Valmayor’ (Mequinenza, Zaragoza). In: I Congreso Internacional de arte rupestre. Caspe, Zaragoza: Bajo Aragon. Prehistoria.; 1986. p. 179–90.

4. Rojo Guerra MA, Tejedor Rodríguez C, Jiménez Jiménez I, Peña Chocarro L, Royo Guillén JI, García-Martínez de Lagrán Í, et al. Releyendo el fenómeno de la neolitización en el Bajo Aragón a la luz de la excavación del Cingle de Valmayor XI (Mequinenza, Zaragoza). Zephyrus: Revista de prehistoria y arqueología. 2015 Jun 15;LXXV:41–71.

5. García Puchol O, Diez Castillo A, Pardo Gordó S. New insights into the neolithisation process in southwest Europe according to spatial density analysis from calibrated radiocarbon dates. Archaeological and anthropological sciences. 2017; doi:10.1007/s12520-017-0498-1

6. Blasco A, Edo M, Villalba MJ, Saña M. Primeros datos sobre la utilización sepulcral de la Cueva de Can Sadurní (Begues, Baix Llobregat) en el Neolítico Cardial. In: III Congreso del Neolítico en la Península Ibérica. Santander: Universidad de Cantabria Servicio de Publicaciones; 2005. p. 625–34.

7. Edo M, Millán M, Blasco A, Blanch M. Resultats de les excavacions de la Cova de Can Sadurní. Tribuna d’Arqueologia. 1985-1986;33–41.

8. Edo M, Blasco A, Millán M, Blanch M. La cova de Can Sadurní. Begues, Baix Llobregat: sis campanyes d’excavació 1978-1983. Memória d’excavació Servei d’Arqueologia. 1991.

9. Blasco A. Les ocupacions prehistòriques a la cova de Can Sadurní (Begues, Baix Llobregat). Memòria de Llicenciatura. Barcelona: Universitat de Barcelona; 1993.

10. Edo M, García Argüelles P, Blasco A, Villalba MJ. La cova de Can Sadurní. Aproximació als primers resultats de la campanya d’excavió de 1993. Cultures i Medi de la Prehistòria a l’Edat Mitjana. 1995;273–84.

11. Blasco A, Edo M, Villalba J, Buxó R, Juan-Tresserras J, Saña M. Del cardial al postcardial en la cueva de Can Sadurní (Begues, Barcelona). Primeros datos sobre su secuencia estratigráfica, paleoeconómica y ambiental. In: II Congrés del Neolític a la Península Ibèrica. Saguntum: Papeles del Laboratorio de Arqueología de Valencia; 1999. p. 59–67.

12. Mestres J. La indústria lítica en sílex del Neolític Antic de les Guixeres de Vilobí. Olerdulae, Revista del Museu de Vilafranca. 1987;3.

13. Mestres J. Avançament a l’estudi del jaciment de Les Guixeres de Vilobí. Pyrenae. 1981;17:35–53.

14. Gibaja Bao JF, Oms FX, Mestres i Mercadé J, Mazzucco N, Palomo A. Primeros resultados sobre la función del utillaje lítico de las primeras comunidades neolíticas asentadas en Les Guixeres de Vilobí (Sant Martí Sarroca, Barcelona). Saguntum: Papeles del Laboratorio de Arqueología de Valencia. 2018;50:35–56.

15. Martínez Fernández G, Afonso Marrero JA, Cámara Serrano JA, Molina González F. Contextualización cronológica y análisis tecnotipológico de los artefactos tallados del Neolítico antiguo de Los Castillejos (Montefrío, Granada). In: Gibaja Bao JF, Faustino Carvalho A, editors. Os últimos caçadores-recolectores e as primeiras comunidades productoras do sul da Península Ibérica e do norte de Marrocos. Faro: Promontoria Monográfica; 2010. p. 163–71.

16. Perales Barrón U, Gibaja Bao JF, Afonso Marrero JA, Martínez Fernández G, Cámara Serrano JA, Molina González F. Análisis funcional del utillaje laminar del Neolítico antiguo de Castillejos de Montefrío (Granada). SPAL - Revista de Prehistoria y Arqueología. 2015;(24):15–33.

17. Arribas A, Molina F. El poblado de ‘los Castillejos’ en las peñas de los gitanos (Montefrío, Granada): campaña de excavaciones de 1971: el corte no. 1. Granada: Secretariado de Publicaciones para el Departamento de Prehistoria de la Universidad de Granada; 1979. 306 p.

18. Afonso Marrero JA, Molina González F, Cámara Serrano JA, Moreno Quero M, Ramos Cordero U, Rodríguez-Ariza MO. Espacio y tiempo. La secuencia en Los Castillejos de Las Peñas de Los Gitanos (Montefrío, Granada). Rubricatum: revista del Museu de Gavà. 1996;1:297–304.

19. Ramos Muñoz J, Afonso Marrero JA, Cámara Serrano JA, Molina González F, Moreno Quero M. Trabajos de acondicionamiento y estudio científico en el yacimiento de Los Castillejos de Las Peñas de Los Gitanos, Granada. In: Anuario Arqueológico de Andalucía. 1997. (III Actividades de Urgencia; vol. III).

20. Cámara Serrano JA, Molina González F, Afonso Marrero JA. La cronología absoluta de Los Castillejos en Las Peñas de los Gitanos (Montefrío, Granada). Cantabria: Universidad de Cantabria; 2005.

21. Martín Socas D, Massieu M, Gonzalez Quintero P. La Cueva de El Toro (Sierra de El Torcal-Antequera-Málaga). Un modelo de Ocupación Ganadera en el Territorio Andaluz entre el VI y II Milenios A.N.E. Consejería de Cultura. Junta de Andalucía. Sevilla; 2004.

22. Camalich Massieu MD, Martín Socas D. Los inicios del Neolítico en Andalucía. Entre la tradición y la innovación. MENGA Revista de Prehistoria de Andalucía. 2013;04:103–29.

23. Rodríguez Rodríguez A, Gibaja JF, Perales Barrón U, Clemente Conte I. Comunidades campesinas, pastoras y artesanas. Traceología de los procesos de trabajo durante el Neolítico andaluz. MENGA Revista de Prehistoria de Andalucía. 2013;4:35–52.

24. Herrero Lapaz N, Pérez Rodríguez M, Lazarich González M, Castañeda Fernández V, García Pantoja ME, Montañés Caballero M, et al. Análisis tecnológico: la industria lítica tallada de ‘El Retamar’. In: Lazarich González M, Ramos Muñoz J, editors. El asentamiento de ‘El Retamar’ (Puerto Real, Cádiz): contribución al estudio de la formación social tribal y a los inicios de la economía de producción en la Bahía de Cádiz. Cádiz: Servicio de Publicaciones; 2002. p. 249–362.

25. Ramos Muñoz J, Lazarich González M. El asentamiento de ‘El Retamar’ (Puerto Real, Cádiz) : contribución al estudio de la formación social tribal y a los inicios de la economía de producción en la Bahía de Cádiz. Cádiz: Universidad de Cádiz. Servicio de Publicaciones; 2002.

26. Ramos Muñoz J, Lazarich González MD, Castañeda Fernández V, Pérez Rodríguez M, Herrero Lapaz N, García Pantoja ME, et al. Modo de producción, modos de vida y valoración socioeconómica de la formación social tribal en el asentamiento de ‘El retamar’ (Puerto Real, Cádiz, España). Revista Atlántica-Mediterránea de Prehistoria y Arqueología Social. 2001;4:115–67.

27. Aura Tortosa JE, Badal E, García Borja P, Garcia-Puchol O, Pascual Benito JL, Jordá Pardo JF. Cueva de Nerja (Málaga). Los niveles neolíticos de la Sala del Vestíbulo. In: III Congreso de Neolítico en la Península Ibérica. Santander; 2005. p. 975–87.

28. Simón Vallejo MD. Una secuencia con mucha prehistoria: la Cueva de Nerja. Mainake. 2003;(25):249–74.

29. Gibaja Bao JF, Cortés Sánchez M, Simón Vallejo MD. La función del utillaje lítico neolítico. El ejemplo de la Cueva de Nerja (Málaga). SPAL - Revista de Prehistoria y Arqueología. 2010;19:97–110.

30. Aura Tortosa JE, Pérez Ripoll M, Jordá Pardo JF, García Borja P, Morales Pérez JV, García Puchol O, et al. Sobre la transición al Neolítico. Las excavaciones Jordá de la Cueva de Nerja (Málaga, España). In: Gibaja Bao JF, Carvalho AF, editors. Os últimos caçadores-recolectores e as primeiras comunidades produtoras do sul da Península Ibérica e do norte de Marrocos. Faro: Promontoria Monográfica; 2010. p. 221–8.

31. Aura Tortosa JE, Jordá Pardo JF, García Borja P, García Puchol O, Badal E, Pérez Ripoll M, et al. Una perspectiva mediterránea sobre el proceso de neolitización. Los datos de la Cueva de Nerja en el contexto de Andalucía (España). MENGA Revista de Prehistoria de Andalucía. 2013;4:53–78.

32. Gimenez PT, Maestre FJJ, Segui EL. Benàmer (Muro d’Alcoi, Alicante): mesolíticos y neolíticos en las tierras meridionales valencianas. Valencia: Diputación de Valencia; 2011.

33. Juan Cabanilles J. El utillaje de piedra tallada en la Prehistoria reciente valenciana: aspectos tipológicos, estilísticos y evolutivos. SIP, Valencia: Diputación de Valencia; 2008. (109).

34. Martí Oliver B, Pascual Pérez V, Gallart Martí MD, Pérez Ripoll M, Acuña Hernández JD, Robles Cuenca F. Cova de l’Or (Beniarrés, Alicante). Vol. II. Valencia: Diputación de Valencia; 1980. (Serie de Trabajos Varios; vol. II).

35. Martí Oliver B. La Cova de l’Or (Beniarrés, Alicante). Saguntum: Papeles del Laboratorio de Arqueología de Valencia. 2011;(Extra 12):183–6.

36. Esquembre Bebiá MA, Boronat Soler J de D, Jover Maestre FJ, Molina Hernández FJ, Luján Navas A, Fernández López de Pablo J, et al. El yacimiento neolítico del Barranquet de Oliva (Valencia). In: IV Congreso del Neolítico Peninsular. 2008. p. 183–90.

37. Bernabeu Auban J, Molina Balaguer L, editors. La Cova de les Cendres. Vol. Serie Mayor, núm. 6. Alicante: MARQ. Museo Arqueológico Provincial de Alicante; 2009.

38. García-Puchol O, Aura Tortosa JE. El abric de la Falguera (Alcoi, Alacant): 8.000 años de ocupación humana en la cabecera del río de Alcoi. Ayuntamiento de Alcoy. Ayuntamiento de Alcoy; 2006.

39. Molina Balaguer L, Bernabeu J, Orozco Köhler T. El Mas d’Is (Penàguila, Alicante). Saguntum: Papeles del Laboratorio de Arqueología de Valencia. 2011;12:179–82.

40. Cortell-Nicolau A. Geomeasure: GIS and Scripting for Measuring Morphometric Variability. Lithic Technology. 2019;44(3):153–65.

41. Cortell-Nicolau A. GeomeasuRe. An R package for capturing morphometric variability. RStudio; 2021. Available: https://github.com/acortell3/GeomeasuRe

42. R Core Team. R: A language and environment for statistical computing. [Internet]. Vienna, Austria.: R Foundation for Statistical Computing; 2020. Available from: https://www.R-project.org/

43. Mitteroecker P, Gunz P. Advances in Geometric Morphometrics. Evolutionary Biology. 2009;36:235–47.

44. Adams DC, Otárola-Castillo E. Geomorph: an R package for the collection and analysis of geometric morphometric shape data. Methods in Ecology and Evolution. 2013;4(4):393–9.

45. Klingenberg CP. Size, shape, and form: concepts of allometry in geometric morphometrics. Development Genes and Evolution. 2016;226:113–37.

46. Claude J. Morphometrics with R. Montpellier: Springer; 2008.

47. Barrière Cl, Daniel R, Deporte H, Escalon de Fonton M, Parent R, Roche AJ, et al. Epipaléolithique-Mésolithique. Les microlithes géométriques. Bulletin de la Société préhistorique française Comptes rendus des séances mensuelles. 1969;355–66.

48. Legendre P. Comparison of permutation methods for the partial correlation and partial mantel tests. Journal of Statistical Computation and Simulation. 2000;67(1):37–73.

49. Guillot G, Rousset F. Dismantling the Mantel tests. Methods in Ecology and Evolution. 2013;4(4):336–44.

50. Legendre P. Should the Mantel test be used in spatial analysis? Methods in Ecology and Evolution. 2015;6(11):1239–47.

51. Isern N, Zilhão J, Fort J, Ammerman AJ. Modeling the role of voyaging in the coastal spread of the Early Neolithic in the West Mediterranean. Proceedings of the National Academy of Sciences. 2017;114(5):897–902.

52. Baselga A, Gómez-Rodríguez C. Assessing the equilibrium between assemblage composition and climate: A directional distance-decay approach. Journal of Animal Ecology. 2021;1–13.

53. Crema ER, Bevan A. Inference from large sets of radiocarbon dates: software and methods. Radiocarbon. 2021;63(1):23–39.

54. Beaumont MA, Nielsen R, Robert C, Hey J, Gaggiotti O, Knowles L, et al. In defence of model-based inference in phylogeography. Molecular Ecology. 2010;19(3):436–46.
